# SMARCD3 Overexpression Promotes Epithelial–Mesenchymal Transition in Gastric Cancer

**DOI:** 10.3390/cancers16122282

**Published:** 2024-06-20

**Authors:** Sun Yi Park, Ji-Ho Park, Jung Wook Yang, Eun-Jung Jung, Young-Tae Ju, Chi-Young Jeong, Ju-Yeon Kim, Taejin Park, Tae-Han Kim, Miyeong Park, Young-Joon Lee, Sang-Ho Jeong

**Affiliations:** 1 Department of Surgery, Gyeongsang National University Hospital, Gyeongsang National University College of Medicine, Jinju 52727, Republic of Koreadrjcy@gnu.ac.kr (C.-Y.J.);; 2 Department of Pathology, Gyeongsang National University Hospital, Gyeongsang National University College of Medicine, Jinju 52727, Republic of Korea; 3 Department of Surgery, Gyeongsang National University Changwon Hospital, Gyeongsang National University College of Medicine, Changwon 51472, Republic of Korea; drjej@gnu.ac.kr (E.-J.J.); thkim@gnuh.co.kr (T.-H.K.); 4 Department of Anesthesiology, Gyeongsang National University Changwon Hospital, Gyeongsang National University College of Medicine, Changwon 51472, Republic of Korea

**Keywords:** gastric neoplasm, biomarker, epithelial–mesenchymal transition, prognosis

## Abstract

**Simple Summary:**

This study explores SMARCD3’s role in gastric cancer, focusing on its elevated expression in signet ring cell (SRC) versus well-differentiated (WD) groups. Elevated SMARCD3 levels in SRC correlated with poorer survival outcomes (HR 2.16, *p* < 0.001), as shown by Kaplan–Meier analysis. Functional assays involving SMARCD3 knock-in and knock-out highlighted that its depletion reduces cell proliferation, migration, invasion, and EMT marker expression, while overexpression increases cell irregularity and area (*p* < 0.001). Further investigations into signaling pathways revealed that SMARCD3 overexpression boosts p-AKT-S473 and p-ERK levels in MKN-74 cells and β-catenin and PI3Kp85 activities in KATO III cells. Conversely, its knock-out decreases these activities in SNU 601 cells. These results suggest that SMARCD3′s overexpression could serve as a negative prognostic marker and a potential target for gastric cancer therapy.

**Abstract:**

This study investigates the role of SMARCD3 in gastric cancer by comparing its expression in signet ring cell (SRC) and well-differentiated (WD) groups within gastric cancer cell lines and tissues. We observed elevated SMARCD3 levels in the SRC group compared to the WD group. Functional analysis was conducted through both SMARCD3 knock-in and knock-out methods. Kaplan–Meier survival analysis indicated that higher SMARCD3 expression correlates with poorer overall survival in gastric cancer patients (HR 2.16, *p* < 0.001). SMARCD3 knock-out cells showed decreased proliferation, migration, invasion, and expression of epithelial–mesenchymal transition (EMT) markers, contrasting with results from temporary and stable SMARCD3 overexpression experiments, which demonstrated increased cell area and irregularity (*p* < 0.001). Further analysis revealed that SMARCD3 overexpression in MKN-74 cells significantly enhanced p-AKT-S473 and p-ERK levels (*p* < 0.05), and in KATO III cells, it increased β-catenin and PI3Kp85 activities (*p* < 0.05). Conversely, these activities decreased in SNU 601 cells following SMARCD3 depletion. The study concludes that SMARCD3 overexpression may serve as a negative prognostic marker and a potential therapeutic target in gastric cancer treatment due to its role in promoting EMT.

## 1. Introduction

Globally, gastric cancer is identified as the fifth most common type of cancer, with approximately 1 million new cases in 2020 [1]. It also ranks fourth in cancer mortality, causing approximately 770,000 deaths that year. In South Korea, gastric cancer is the leading type of cancer among men and the fourth most common cause of cancer death. The Korea Central Cancer Registry data from 2021 show that 29,361 people were newly diagnosed with gastric cancer, accounting for 10.6% of all cancer cases in the country [2].

In the last ten years, the increasing incidence of gastric cancer has shifted its impact from affecting only individuals to affecting families and the wider community. The treatment for this disease is extensive and intricate, requiring cooperation from various specialties within medicine. Surgery is the primary method of treatment for gastric cancer, but recent technological advancements have led to the adoption of new methods such as robotic surgery, genetic testing, immunotherapy, and tailored treatments. On a global scale, gastric cancer is acknowledged as a major health challenge, with increasing rates observed in several countries due to factors such as changing demographics, dietary patterns, and a reduction in infectious diseases [3,4,5].

Epithelial–mesenchymal transition (EMT), a complex process by which epithelial cells adopt the properties of mesenchymal cells, plays a pivotal role in the progression of cancer [6]. In cancer, EMT is associated with tumorigenesis, invasion, metastasis, and resistance to treatment, making it difficult to treat. E-cadherin is a cell adhesion molecule that is crucial for maintaining the integrity of epithelial tissues. Loss of E-cadherin expression is a hallmark of EMT and is associated with increased invasiveness. EMT often involves the downregulation of E-cadherin, leading to reduced cell-cell adhesion and increased motility of cancer cells [7,8,9].

E-cadherin plays a crucial role in multiple signaling pathways involved in carcinogenesis, including the WNT, Hippo, RTK, growth factor, and GTPase pathways [10,11,12,13]. Among these pathways, beta-catenin, PI3K, and MAPK are known to translocate to the nucleus, indicating their involvement in downstream signaling events [14,15]. Moreover, the localization-dependent responses to EGF ligands and growth factors contribute to various tumor development pathways. The AKT pathway is a signal transmission pathway related to EMT, and AKT phosphorylates GSK3b-S9 and inactivates GSK to stabilize β-catenin by binding to Axin, APC, and GSK3b-Tyr216, which accumulates in the cytoplasm. After that, it moves into the nucleus and activates genes related to transcription due to TCF/LEF and the complex, which upregulates genes related to proto-oncogenes (c-Myc, cyclin D1) [16,17,18,19,20,21,22,23]. It has also been reported that SMARCD3 in breast and colorectal cancer cells is regulated by the activation of the WNT signaling pathway [24,25].

In previous studies [26], we analyzed the transcriptomes (gene expression profiles) of two distinct types of gastric cancer: poorly cohesive carcinoma (PCC), also known as diffuse-type, and well-differentiated tubular adenocarcinoma (WD), also referred to as intestinal-type gastric cancer. This analysis was carried out using both gastric cancer tissues and cell lines. Our findings indicated that SMARCD3 levels were elevated in SRC compared to WD in both gastric cancer cell lines and tissues. SMARCD3, a member of the SWI/SNF chromatin remodeling complex, is integral to the regulation of chromatin structure and gene expression. As a subunit of this complex, SMARCD3 plays a pivotal role in chromatin remodeling and controlling gene expression. The function of SMARCD3 and the SWI/SNF complex in cancer is an active area of research, with alterations observed in various cancer types [27,28]. The role of SMARCD3 differs across cancer types. In pancreatic cancer, SMARCD3 is linked to a poor prognosis [29], while in uterine and breast cancers, it functions as a tumor suppressor [30]. However, its role in gastric cancer has not been thoroughly investigated. This study aimed to elucidate the role of SMARCD3 in gastric cancer.

## 2. Materials and Methods

### 2.1. Cell Lines and Transfection

Human gastric cell lines MKN45, MKN74, SNU601, SNU668, and KATO III were obtained from the Cell Line Bank in Seoul, Korea. These cell lines were cultured in RPMI 1640 medium (Gibco, Thermo Fisher Scientific, Inc., Waltham, MA, USA), a nutrient-rich solution that supports the growth and maintenance of these cells. The medium was supplemented with 10% heat-inactivated fetal bovine serum (FBS, Gibco, Thermo Fisher Scientific, Inc.), which provides essential growth factors, hormones, and nutrients necessary for cell proliferation and survival. Additionally, penicillin (100 U/mL, Thermo Fisher Scientific, Inc.) was included to prevent bacterial contamination, ensuring a sterile environment for cell growth. The cells were maintained at a constant temperature of 37 °C in a humidified incubator with 5% CO_2_, mimicking the physiological conditions of the human body to promote optimal cell growth and function.

For genetic modification, the cells were transfected with two types of plasmid vectors. The first vector used was the pCMV6 control vector (cat#: PS100001, Rockville, MD, USA), which serves as a baseline for comparison in experiments. The second vector was the pCMV6-SMARCD3-FLAG-DDK-tagged expression construct vector (cat#: RC222004, Rockville, MD, USA), specifically designed to overexpress the SMARCD3 gene, tagged with FLAG and DDK for easy identification and purification. Transfection was carried out using Lipofectamine 2000 (Invitrogen, Waltham, MA, USA), a widely used reagent that facilitates the delivery of DNA into cells by forming lipid–DNA complexes that merge with the cell membrane, allowing the DNA to enter the cells efficiently. This method is essential for studying gene function and protein expression in the context of gastric cancer research, providing insights into the molecular mechanisms underlying this disease. To downregulate SMARCD3, SNU601 and SNU668 cells were transfected with negative control siRNA (#4390844, Ambion, Austin, TX, USA) and SMARCD3 siRNA (#4392420, s13158 and #439240, s13159 Ambion, Austin, TX, USA) using Lipofectamine Maxi Reagent (Invitrogen). Stable cell lines were created in MKN45 and MKN74 cells, treated with 700 µg/mL or 450 µg/mL G418, and selected for colonies. Techniques used included Western blotting, cell proliferation assays, Transwell migration and invasion assays, and analysis of epithelial–mesenchymal transition markers [31].

### 2.2. Western Blotting

Cells were lysed using RIPA buffer (Thermo Fisher Scientific, Inc. #89901), a commonly used lysis buffer designed to effectively break down cell membranes and release cellular proteins. This buffer was supplemented with protease inhibitors (Sigma, St. Louis, MO, USA, P8340), which prevent the degradation of proteins by inhibiting the activity of proteases, and phosphatase inhibitors (Thermo Fisher Scientific, Inc. 78420), which prevent the removal of phosphate groups from proteins, thereby preserving their phosphorylation state. This careful preparation ensures that the proteins remain intact and functional for subsequent analysis. Following lysis, 20 μg of protein from each sample was separated using a 10% SDS-PAGE gel via electrophoresis. SDS-PAGE (sodium dodecyl sulfate–polyacrylamide gel electrophoresis) is a technique that allows proteins to be separated based on their molecular weight. The separated proteins were then transferred to PVDF membranes using the iBlot 2 PVDF Regular Stacks system (Invitrogen, Thermo Fisher Scientific, Inc.), which ensures efficient and consistent protein transfer from the gel to the membrane.

To prevent nonspecific binding of antibodies, the PVDF membranes were blocked for 1 h at room temperature with 5% skim milk. This blocking step is crucial as it coats the membrane with a protein that can occupy any potential nonspecific binding sites. The membranes were then incubated overnight at 4 °C with primary antibodies specific to the target proteins: anti-E-cadherin (Cell Signaling #14472, Danvers, MA, USA), anti-SMARCD3 (Cell Signaling #62265), anti-Snail (Abcam ab216347, Waltham, MA, USA), anti-Slug (Abcam ab51772), and anti-GAPDH (Santa Cruz Biotechnology sc-47724, Dallas, TX, USA). These primary antibodies bind specifically to their respective target proteins, allowing for precise detection. After the overnight incubation, the membranes were washed three times with TBST/Tween 20 buffer to remove any unbound primary antibodies. The membranes were then incubated with a secondary antibody for 1 h at room temperature. The secondary antibody is designed to bind to the primary antibody and is usually conjugated with a detection enzyme or fluorophore. Finally, protein detection was performed using an enhanced chemiluminescence (ECL) system (Bio-Rad Laboratories, Inc., Hercules, CA, USA). The ECL system produces a luminescent signal when the enzyme on the secondary antibody catalyzes a reaction, allowing for the visualization and quantification of the target proteins on the membrane. This method provides a sensitive and accurate means to detect specific proteins within the complex mixture of cellular lysates, making it a powerful tool in molecular biology and biochemistry research.

### 2.3. Proliferation Assay

Cells (2 × 10^4^ cells per well) were seeded in 24-well plates. Following seeding, MKN74 and KATO III cells were transfected to overexpress pCMV6-entry and pCMV6-SMARCD3. To knock down SMARCD3, SNU601 and SNU668 cells were transfected with control siRNA, SMARCD3 siRNA #1, or SMARCD3 siRNA #2. During incubation, MTT (3-(4,5-dimethylthiazol-2-yl)-2,5-diphenyltetrazolium bromide) reagent was added to each well. After incubation, dimethyl sulfoxide (DMSO) was added to each well to dissolve the formazan crystals. The optical density (OD) of the resulting solution was measured at 570 nm using a microplate reader. Measurements were taken at different time points: 0, 24, 48, and 72 h after the start of culture.

### 2.4. Colony Formation Assay

Transfected MKN74, KATO III, SNU601, and SNU668 cells (300 cells per well) were seeded in 6-well plates and incubated at 37 °C for 10–14 days, with the medium changed every three days. The cells were rinsed with PBS, fixed in 100% methanol at 4 °C for 20 min, rinsed with PBS again, and stained with crystal violet (1% *w*/*v*; Sigma-Aldrich; Merck, Darmstadt, Germany) for 1–3 h at room temperature. The samples were scanned using a UMAX PowerLook 2100XL scanner (MagicScan software, version 4.5, UMAX) and then analyzed with ImageJ (version 1.52d, NIH).

### 2.5. Wound-Healing Migration Assay

Gastric cancer cell lines (MKN74, KATO III, SNU601, and SNU668) were seeded at a density of 1 × 10^5^ cells per well in 70 µL of media in Culture-Insert 2 wells (Ibidi, GmbH, Gräfelfing, Germany) within 6-well plates. To reduce cell proliferation, mitomycin C (10 µg/mL) was added 2 h before removing the inserts. After cells adhered, inserts were removed to create cell-free gaps, and the media was replaced. Photomicrographs were taken immediately (time 0 h) and after incubation at 37 °C for 24 h to 6 days to assess wound closure and cell migration. Migration was quantified by counting migrated cells using NIH ImageJ software (version 1.52d, NIH). Data are presented as the mean ± SD from three independent experiments, with microscopy images taken at ×40 magnification.

### 2.6. Invasion Assay

The detailed experimental methods for Western blotting, proliferation assays, Transwell migration assays, invasion assays, and epithelial–mesenchymal transition marker assays are documented in previous reports. Transwell chambers with 8 μm pores (Corning Inc., Kennebunk, ME, USA) and Transwell membranes precoated with Matrigel (2.2 mg/mL) were used. MKN74, KATOIII, SNU601, and SNU668 cells were harvested and suspended in serum-free RPMI medium at a concentration of 1 × 10^5^ cells/mL and seeded into the upper chambers of a 24-well plate. The lower chambers were filled with RPMI medium containing 10% FBS. After the experiment, cells that had migrated or invaded the underside of the Transwell membrane were fixed with 4% formalin and stained with 4′,6-Diamidino-2-phenylindole (DAPI, Sigma-Aldrich; Merck KGaA). The cells were counted under an inverted light microscope, and images of invasive cells were analyzed using NIH ImageJ software (version 1.52d, NIH). Data are presented as the mean ± SD from three independent experiments, with microscope images taken at ×200 magnification.

### 2.7. Kaplan-Meier Survival Curve Analysis

Kaplan–Meier analysis (KM plotter, https://kmplot.com/analysis/index.php?p=service&cancer=gastric (accessed on 27 May 2022)) is a statistical method used to estimate the survival function from data containing information about the time to an event (e.g., death) for different groups. The dataset included Affymetrix array data, and the analysis focused on the SMARCD3 gene probe set (204099_at). All data were derived from the 204099_at set database. The color of the survival curve in the Kaplan-Meier plot indicates the gene expression level of SMARCD3 (red: high expression, black: low expression). The number of subjects at risk (indicating the number of individuals still at risk of experiencing the event at each time point), hazard ratio (HR), 95% confidence intervals (CIs), and log-rank *p* values are provided on the webpage. A significance threshold of *p* < 0.05 was considered statistically significant.

### 2.8. Microscopic Examination

Using microscopy, we observed changes in cell behavior following SMARCD3 expression. Cell size was measured at three or more randomly selected locations in both transiently expressing and stably overexpressing MKN74 cells. Measurements were taken within a size range of 50 to 100 μm using an optical microscope (NIS-Elements BR 3.2, Nikon Eclipse Ti-S, Nikon, Tokyo, Japan). ImageJ software (version 1.52d, NIH) was used to calculate cell areas in both the control group and the SMARCD3-overexpressing group. Microscope images were taken at ×200 magnification.

### 2.9. Immunocytochemistry

Transfected MKN74 cells were fixed in 4% paraformaldehyde in PBS for 10 min. The fixed cells were permeabilized with 0.5% Triton X-100 for 10 min. After permeabilization, the cells were blocked with 5% BSA in PBS for 1 h and then incubated with the appropriate primary antibodies. The primary antibodies [mouse anti-E-cadherin (Cell Signaling #14472, MA, USA, 1:300 in PBS, 5% BSA) and rabbit anti-SMARCD3 (Cell Signaling #62265, MA, USA, 1:300 in PBS, 5% BSA)] were incubated with the cells overnight at 4 °C. Following this, the cells were washed three times with PBS and incubated with a fluorescence-conjugated secondary antibody for 1 h. After incubation, the secondary antibody was washed away. Nuclei were stained with ProLong Gold antifade reagent with DAPI (p36934, Invitrogen, Eugene, OR, USA). For image acquisition, a Nikon Eclipse Ti confocal microscope system (Nikon Instrument, Inc.) was used. Microscope images were taken at ×400 magnification.

### 2.10. Statistical Analysis

BM^®^ SPSS^®^ Statistics version 27 software (IBM Corp., Armonk, NY, USA) was used for statistical analysis. The significance of differences was determined using the χ^2^ test, which is a statistical test used for categorical data. Student’s *t* test was used for analyzing continuous variables between two groups, and ANOVA test was used for analyzing continuous variables between more than 3 groups (Supplementary, Figures S1–S10). Kaplan-Meier’s method, a survival analysis technique, was used to analyze patient outcomes and overall survival. GRAPHPAD Prism 7.0 software (GraphPad Software, Inc., San Diego, CA, USA) was used for in vitro analysis. The significance threshold for determining statistically significant differences was set at *p* < 0.05.

### 2.11. Ethics Statement

The studies were designed and carried out in accordance with the principles of the Declaration of Helsinki (2013 revision). Approval was obtained from the Institutional Review Board (GNUHIRB 2009-54). Patient consent was waived as there was no identifiable information related to the patients.

## 3. Results

### 3.1. SMARCD3 Was More Highly Expressed in Gastric Cancer Tissues Than in Normal Tissues, Especially in SRCs

Following our previously reported NGS analysis [31], we confirmed that the SMARCD3 gene is overexpressed in SRC tissues and cells compared to WD tissues and cells. This was observed in three SRC-GC and five WD-GC samples obtained in a previous study, along with data from newly analyzed gastric cancer cell lines: SRC-GC (KATO III, SNU601) and WD-GC (MKN 74-1, 74-2) [26].

**Figure 1:**
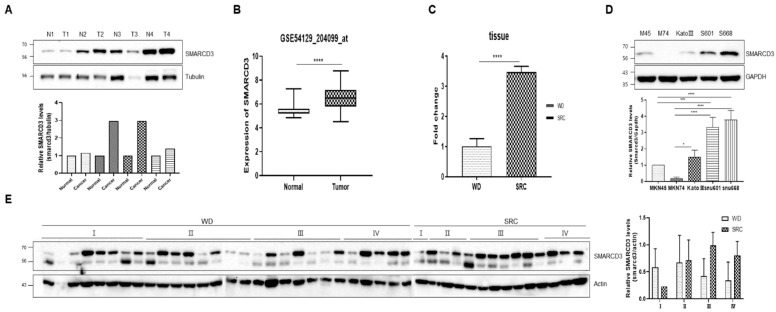
SMARCD3 expression in tissue and cell-line. (**A**) SMARCD3 expression increased in tumor tissues compared to normal tissues in the same-stage patients in gastric tissue analysis. (**B**) GSE 54129 mRNA microarray analysis showed that SMARCD3 mRNA expression was significantly higher in cancer tissues than in normal tissues. (**C**) SMARCD3 expression was increased in the SRC group compared to the WD group in previously reported NGS analysis. (**D**) Comparison of SMARCD3 expression in gastric cancer cell lines using immunoblot. (**E**) Comparison of SMARCD3 expression in TNM stage gastric cancer patients: well-differentiated gastric cancer (WD-GC) and signet ring cell gastric cancer (SRC-GC). Statistical analysis was performed by one-way ANOVA. * *p* < 0.05, *** *p* < 0.001, and **** *p* < 0.0001.

A comparison of normal and tumor tissues revealed that SMARCD3 expression was higher in tumor tissues than in normal tissues in patients with the same stage of gastric cancer (TNM stage III) (Figure 1A and Figure S11). Similar findings were reported by Hippo Y et al. in 2002 [32], and GSE 54129 mRNA microarray analysis showed that SMARCD3 mRNA expression was significantly higher in cancer tissues than in normal tissues (Figure 1B) [33].

According to the subgroup analysis, SMARCD3 expression in tissues was higher in the SRC group (3.464 ± 0.19) compared to the WD group (1.013 ± 0.25, **** *p* < 0.0001) (Figure 1C).

The protein expression of SMARCD3 was assessed in gastric cancer cell lines. MKN45 showed a significant difference in expression compared to SNU601 and SNU668 (*** *p* = 0.0002, **** *p* < 0.0001) but not compared to KATOIII. MKN74 cells exhibited significantly different expression levels from KATOIII, SNU601, and SNU668 cells (* *p* = 0.0108, *** *p* < 0.0001, and **** p < 0.0001) (Figure 1D and Figure S12). In gastric cancer tissues, stage I patients with WD-GC had increased SMARCD3 expression, while stage III and IV SRC-GC patients showed higher SMARCD3 expression compared to stage III and IV WD-GC patients (Figure 1E and Figure S13).

### 3.2. The High-SMARCD3 Expression Group Had Poor Overall Survival according to Kaplan-Meier Plotter Data

**Figure 2:**
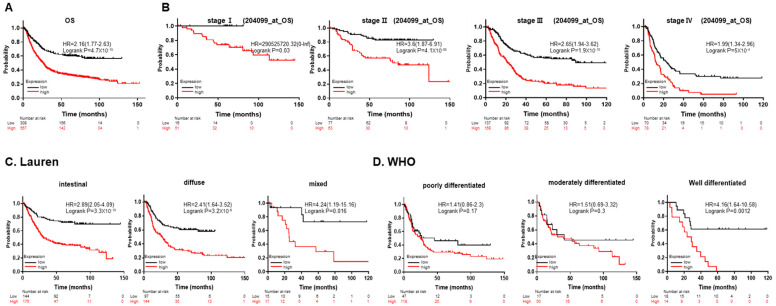
Kaplan-Meier survival curves of patients stratified by SMARCD3 expression. (**A**) OS in all patients and patients in the following groups: (**B**) stage I~IV, (**C**) intestinal, diffuse, and mixed according to the Lauren classification, and (**D**) poorly, moderately, and well-differentiated according to the WHO classification.

Overall, patients with high SMARCD3 expression had a significantly lower overall survival (OS) rate than patients with low SMARCD3 expression (HR 2.16, *p* = 4.7 × 10^−15^; Figure 2A). In patients with TNM stages I~IV, there was a significant difference in OS between the low- and high-expression groups. Higher SMARCD3 expression was associated with significantly lower survival (stage I, II, III, and VI; log-rank test: *p* = 0.03, *p* = 4.1 × 10^−5^, *p* = 1.9 × 10^−10^, *p* = 5 × 10^−4^; Figure 2B).

According to the Lauren classification (intestinal, diffuse, mixed), increased SMARCD3 expression was associated with decreased survival: intestinal type (HR = 2.89; log-rank test: *p* = 3.3 × 10^−10^), diffuse type (HR = 2.41; log-rank test: *p* = 3.2 × 10^−6^), and mixed type (HR = 4.24; log-rank test: *p* = 0.016) (Figure 2C). When we analyzed the association between SMARCD3 expression and OS according to the WHO differentiation classification, we found that in the well-differentiated group, increased SMARCD3 expression was associated with decreased OS (HR = 4.16; log-rank test: *p* = 0.0012). OS in the poorly differentiated subgroup (HR = 1.41; log-rank test: *p* = 0.17) and moderately differentiated subgroup (HR = 1.51; log-rank test: *p* = 0.3) was also worse in the overexpression group, but the difference was not significant (Figure 2D). These results indicated that a high expression of the SMARCD3 gene was associated with poorer overall survival in gastric cancer patients, especially in subgroups according to TNM staging, Lauren classification, and WHO differentiation classification.

### 3.3. SMARCD3-Overexpressing Gastric Cancer Cell Lines Exhibit Increased Migration, Invasion, and EMT Markers

**Figure 3:**
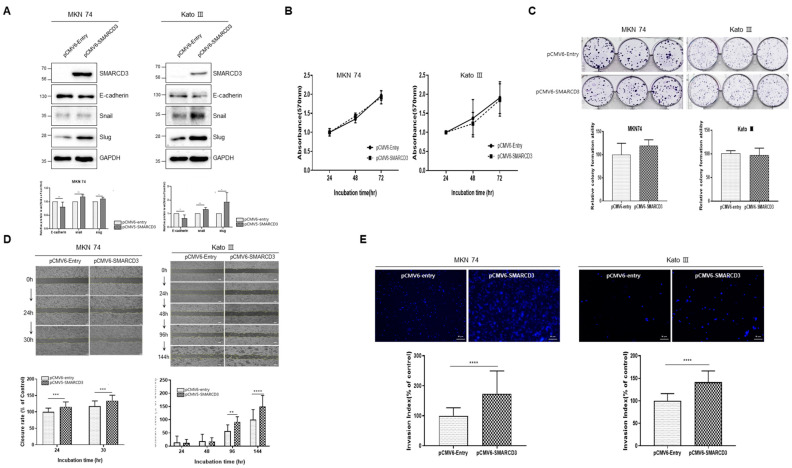
Functional study of SMARCD3-overexpressing MKN74 and KATOIII cell lines. (**A**) Changes in the expression of snail and slug, which are markers of EMT, in SMARCD3-overexpressing MKN74 and Kato III cells were observed via Western blotting. Data graphs of the expression of E-cadherin, snail, and slug, which are markers of EMT. (**B**) proliferation assay. (**C**) colony formation assay. (**D**) wound-healing migration assay (×40 magnification) (**E**) Transwell invasion assay (×200 magnification). Data are presented as the mean ± SD from three independent experiments. Statistical analysis was performed by Student’s *t* test (**A**,**C**,**E**) or two-way ANOVA (**B**,**D**). ** *p* < 0.01, *** *p* < 0.001, and **** *p* < 0.0001.

MKN74 and KATOIII (a gastric cancer cell line with low SMARCD3 expression) were transfected with pCMV6-SMARCD3, and SMARCD3 overexpression was confirmed by Western blot (Figure 3A and Figure S14).

We evaluated the expression of epithelial–mesenchymal transition (EMT) markers (E-cadherin, Snail, and Slug). MKN 74 cells exhibited decreased E-cadherin (** *p* = 0.0027) and upregulated snail (** *p* = 0.0036) and slug (* *p* = 0.0242) after overexpression (Figure 3A). In KATOIII cells, E-cadherin expression (* *p* = 0.0146) was decreased, and snail expression (** *p* = 0.0041) and slug expression (* *p* = 0.0469) were significantly increased (Figure 3A and Figure S15).

According to the proliferation and colony formation assays, the number of cells in the pCMV6-SMARCD3 GC group did not significantly increase compared to that in the pCMV6-Entry GC group (Figure 3B,C). In the wound-healing assay, MKN74 cells showed a significant increase in pCMV6-SMARCD3 GCC compared to pCMV6-Entry GCC from 24 to 30 h (24 h *** *p* = 0.0009, 30 h *** *p* = 0. 0002, Figure 3D and Figure S1). KATOIII was significantly increased at 96 h and 144 h (** *p* = 0.0088 at 96 h, **** *p* < 0.0001 at 144 h), but it was not different at 24 and 48 h (Figure 3D and Figure S2). According to the invasion assay, the number of pCMV6- ± SMARCD3 GCCs was significantly greater than that of pCMV6-Entry GCCs in MKN74 and KatoIII cells after 48 h (MKN 74; **** *p* < 0.0001, KATO III; ****p < 0.0001) (Figure 3E).

### 3.4. SMARCD3-Knock-Out Gastric Cancer Cell Lines Exhibited Decreased Proliferation, Migration, Invasion and EMT

**Figure 4:**
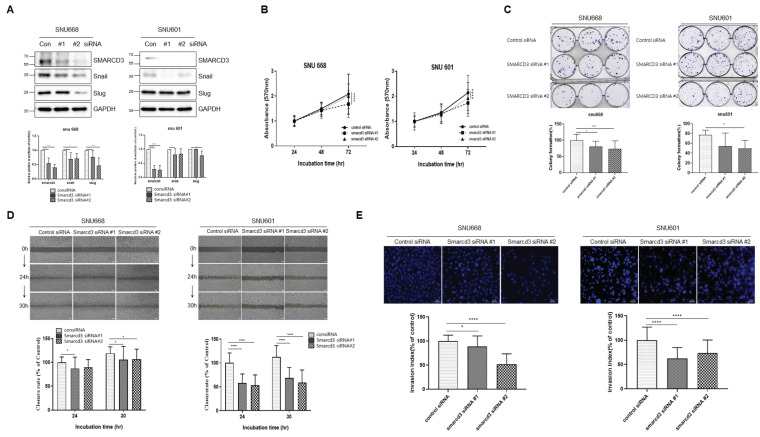
Functional study of the SNU601 and SNU668 cell lines with SMARCD3 knockdown. Changes in the expression of snail and slug, which are markers of EMT, in SNU601 and SNU668 cells with SMARCD3 depletion were observed via Western blotting. Data graphs of the expression of snail and slug, which are markers of EMT (**A**), proliferation (**B**), colony formation (**C**), wound-healing, migration (**D**), and Transwell invasion (**E**) assays. Statistical analysis was performed by two-way ANOVA (**A**,**B**,**D**) or one-way ANOVA (**C**,**E**). * *p* < 0.05, ** *p* < 0.01, *** *p* < 0.001, and **** *p* < 0.0001.

To further confirm that SMARCD3 affects EMT, we investigated EMT by knock-out SMARCD3 in the SNU 668 and SNU 601 cell lines, which have relatively high expressions of SMARCD3. SNU668 and SNU601 (gastric cancer cell lines with high SMARCD3 expression) were knocked out with control siRNA in the control group and with SMARCD3 siRNA #1 and SMARCD3 siRNA #2, respectively (Figure 4A). The SMARCD3 protein level percentage of SNU 668 cells was reduced by more than 60% and 50% with SMARCD3 siRNAs #1 and #2, respectively, and the percentage of SNU 601 cells was reduced by more than 70% with SMARCD3 siRNAs #1 and #2, respectively.

In the SNU 668 cell line, snail expression was significantly lower in the SMARCD3 siRNA#1 and SMARCD3 siRNA#2 groups than in the control siRNA groups (#1 ** *p* = 0.0088, #2 * *p* = 0.0120). Slugs were also significantly reduced in SNU 668 cells transfected with SMARCD3 siRNAs #1 and #2 (#1 * *p* = 0.0433, #2 **** *p* < 0.0001) (Figure 4A, Figures S3 and S16).

In the SNU 601 cell line, snail expression was significantly lower in the SMARCD3 siRNA#1 group than in the control group, but that in the SMARCD3 siRNA#2 group did not decrease significantly (#1 * *p* = 0.014). In the case of slug, the expression of SMARCD3 siRNA#2 was significantly decreased, but the expression of SMARCD3 siRNA #1 was not different (#2 * *p* = 0.014) (Figure 4A, Figures S4 and S17).

In the proliferation assay, a statistically significant reduction in the proliferation of SNU668 and SNU601 cells was observed after treatment with SMARCD3 siRNA #1 (**** *p* < 0.0001) compared to treatment with control siRNA for 72 h (Figure 4B, Figures S5 and S6). In the colony formation assays, the number of SNU668 cells in the SMARCD3 siRNA #1 and #2 groups was significantly lower compared to the control siRNA group; * *p* = 0.014 and *** *p* < 0.0002, respectively. Similarly, the number of SNU601 cells in the SMARCD3 siRNA #2 group was significantly lower than in the control siRNA group (* *p* = 0.025) (Figure 4C).

Migration assays showed that the numbers of SNU601 and SNU668 cells were significantly reduced at 24 and 30 h, respectively (SNU601 SMARCD3 siRNA#1, *p* < 0.0001: siRNA#2, *p* < 0.0001 at 24 h, 30 h; SNU668 SMARCD3 siRNA#1, *p* = 0.019 at 24 h: siRNA#1, *p* = 0.013 siRNA#2, *p* = 0.033 at 30 h) (Figure 4D, Figures S7 and S8). Invasion assays revealed a decrease in the number of SNU668 cells in the SMARCD3 siRNA#1 and siRNA#2 groups (#1 * *p* = 0.014, #2 **** *p* < 0.0001), and a significant decrease in the number of SNU601 cells (SMARCD3 siRNA#1, *p* < 0.0001: siRNA#2, *p* < 0.0001) (Figure 4E, Figures S9 and S10).

The overexpression of SMARCD3 in cells with low SMARCD3 levels accelerated EMT, while the downregulation of SMARCD3 in cells with high SMARCD3 levels tended to reduce EMT. These results suggest that SMARCD3 is closely related to EMT.

### 3.5. Macroscopic Changes after SMARCD3 Overexpression Revealed Irregular Shapes and Increased Average Cell Areas

**Figure 5:**
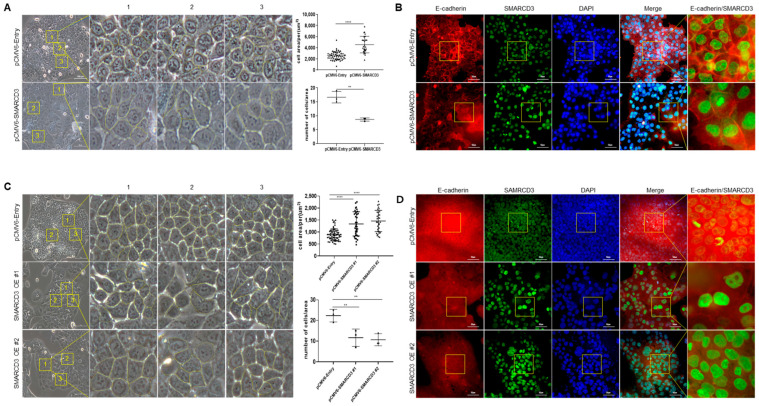
Morphology of MKN74 cells overexpressing SMARCD3. Microscopy was used to compare the shape of the cells (**A**), and fluorescence microscopy was used to analyze the shape of E-cadherin (**B**). The shape of a stable cell line overexpressing SMARCD3 in MKN74 cells (**C**) and the shape of E-cadherin in the stable cell line (**D**). Statistical analysis was performed by Student’s *t*-test (**A**) or one-way ANOVA (**C**). ** *p* < 0.01, and **** *p* < 0.0001. (**A**,**C**) are at 200× magnification, (**B**,**D**) are at 400× magnification, and the yellow box in (**A**,**C**) represents 100 µm on one side.

Using microscopy, we observed changes in cell morphology upon SMARCD3 expression. Both the transiently overexpressing MKN74 cells (Figure 5A,B) and the stably overexpressing MKN74 cells (Figure 5C,D) had irregular and enlarged shapes compared to those of the control cells. Three areas (yellow box, Figure 5A, 100 µm × 100 µm; Figure 5C, 50 µm × 50 µm) were randomly measured and compared using an optical microscope.

In the temporary overexpression experiment, the average area of the SMARCD3-overexpressing group (628.36 ± 215.53 µm^2^) was significantly greater (**** *p* < 0.0001) than that of the control group (378.96 ± 91.20 µm^2^). When the number of cells of the same size (yellow box) was counted, the number of cells was greater in the control group (*n* = 16.6 ± 1.7), the number of cells was lower, and the shape of the cells was irregular (#1, *n* = 8.6 ± 0.4) (** *p* = 0.003) (Figure 5A). To determine the association of cell shape changes with E-cadherin, a hallmark of EMT, we stained for E-cadherin. The shape of the E-cadherin signal was more irregular in the transient SMARCD3-overexpressing group than in the control group (Figure 5B).

Compared with the control group, the stable SMARCD3-overexpressing group (#1; 151.7 ± 147.48 µm^2^, **** *p* < 0.0001; #2; 70.58 ± 14.9 µm^2^, **** *p* < 0.0001) exhibited irregular shapes and increased average cell area (30.7 ± 8.04 µm^2^). When the number of cells of the same size (yellow box) was counted, the number of cells was greater in the control group (*n* = 22.3 ± 2.4), the number of cells was lower, and the shape of the cells was irregular (#1, *n* = 11.6 ± 3.4; #2, *n* = 10.6 ± 2.3) (**** *p* < 0.0001) (Figure 5C).

MKN74 cells stably overexpressing SMARCD3 were examined under a microscope to observe cell shape (Figure 5C) and the appearance of E-cadherin (Figure 5D), as described in a previous study. The results were the same in transiently overexpressing MKN74 cells and stable MKN74 cells. Based on the above results, we found that SMARCD3 is involved in EMT.

### 3.6. Investigation of Cancer Signaling Pathways Using SMARCD3 Overexpression and Knockdown

We investigated the Wnt (b-catenin, GSK), PI3-AKT (AKT), and MAPK (pJNk, ERK, p38) signaling pathways through the increase and decrease in the expression of each gene to determine the submechanism of cancer involving SMARCD3.

**Figure 6:**
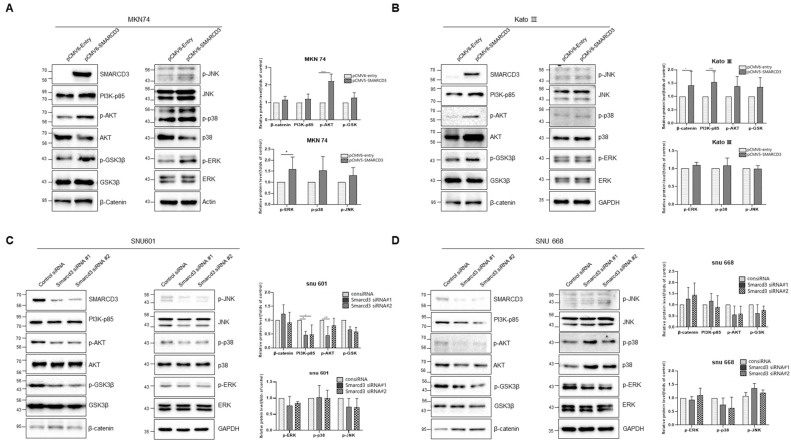
Cancer signaling pathway investigation using overexpression and knockdown of SMARCD3 (Wnt, PI3-AKT, and MAPK pathway). (**A**) Overexpression study in MKN 74 cells. (**B**) Overexpression study in KATO III. (**C**) Knockdown study in SNU 601. (**D**) Knockdown study in SNU 668. Data are presented as the mean ± SD for triplicate independent experiments, and the error bars indicate SD. Statistical analysis was performed by two-way ANOVA(A). * *p* < 0.05, *** *p* < 0.001, and **** *p* < 0.0001.

In the overexpression study, the activities of pAKT-s473 (**** *p* < 0.0001) and p-ERK (* *p* < 0.005, *p* = 0.037) were significantly increased in MKN 74 cells (Figure 6A and Figure S18). In KATO III cells, the activities of β-catenin were increased (* *p* = 0.014) after SMARCD3 overexpression (Figure 6B and Figure S19).

siRNA-mediated knockdown decreased the activity of AKT-s473 (siRNA #1 *** *p* < 0.0001) in SNU 601 cells (Figure 6C and Figure S20). At SNU 668, the AKT-S473 and p-GSK levels decreased (Figure 6D and Figure S21).

The expression of SMARCD3 is involved in Wnt and MAPK signaling, as well as the PI3K-AKT pathway. In cancer-associated processes, not only one signal transmission but also several other signal transmission processes affect cell transmission.

## 4. Discussion

The aim of this study was to determine the role of SMARCD3 in gastric cancer. Compared with those in WD patients, SMARCD3 levels in SRC patients were found to increase, which was correlated with a decrease in survival rates as the TNM stage advanced. This phenomenon was associated with alterations in cell morphology, enhanced epithelial–mesenchymal transition (EMT), heightened metastatic potential, and consequently, poorer prognosis. Further analysis revealed the involvement of several signaling pathways as contributing submechanisms in this process.

SMARCD3, also known as BAF60C, is a member of the SWI/SNF chromatin remodeling complex and has been implicated in various cancers, exerting diverse roles depending on the specific cancer type [27,28,29,30]. In pancreatic cancer, SMARCD3 has been associated with a poor prognosis. Its expression is elevated, correlating with disease progression and metastasis [29]. Mechanistically, SMARCD3 has been linked to promoting tumor growth and invasion in pancreatic cancer cells. Conversely, in uterine and breast cancers, SMARCD3 acts as a tumor suppressor [28,30,34]. Studies have shown that loss of SMARCD3 expression is associated with aggressive tumor behavior, increased metastasis, and poorer patient outcomes. In these cancers, SMARCD3 likely functions to regulate gene expression and maintain genomic stability, thereby inhibiting tumor progression [24,34].

SMARCD3 has not been well studied in gastric cancer. Previously, we compared SRC and WD, and we identified SMARCD3 as a cancer candidate gene [35]. SRC is a specific subtype of gastric cancer characterized by its distinctive appearance under a microscope. These cancer cells have a signet ring-like appearance due to the accumulation of mucin within the cell. Compared with other subtypes, SRC-GC is often more aggressive and has a poorer prognosis. EMT is a process in which epithelial cells lose their characteristics and acquire traits typical of mesenchymal cells [36]. The loss of E-cadherin expression is a hallmark of EMT and is associated with increased invasiveness. EMT often involves the downregulation of E-cadherin, leading to reduced cell-cell adhesion and increased motility of cancer cells [13,34].

The overexpression of SMARCD3 might play a role in promoting EMT in signet ring cell gastric cancer. This promotion of EMT could lead to the downregulation of E-cadherin, contributing to the invasive behavior of cancer cells [8,36]. These combined effects could result in a poorer prognosis for patients with signet ring cell gastric cancer [35]. In our study, we confirmed changes in the morphology of gastric cancer cell lines and alterations in EMT markers when the SMARCD3 gene was overexpressed. Using light microscopy and immunofluorescence staining, we observed the appearance of E-cadherin. The results showed that the nuclei of cells overexpressing SMARCD3 were larger than those of the control cells, and the density and structure of E-cadherin were compromised. These changes may have influenced the EMT of gastric cancer and, consequently, the patient’s prognosis. The expression of SMARCD3 is involved in Wnt and MAPK signaling, as well as the PI3K-AKT pathway. In cancer-associated processes, not only one signal transmission but also several other signal transmission processes affect cell transmission [8,13,25].

E-cadherin plays a crucial role in multiple signaling pathways involved in carcinogenesis, including the WNT, Hippo, RTK, growth factor, and GTPase pathways [29]. Among these pathways, beta-catenin, PI3K, and MAPK are known to translocate to the nucleus, indicating their involvement in downstream signaling events [37]. Moreover, the localization-dependent responses to EGF ligands and growth factors contribute to various tumor development pathways. It has been reported that SMARCD3 in breast cancer cells is regulated by the activation of the WNT signaling pathway [34]. In line with these findings, the authors confirmed the activation of the beta-catenin and AKT signaling pathways through Western blot analysis in cell lines in which SMARCD3 was overexpressed. Specifically, the overexpression of SMARCD3 in MKN74 and KATO3 cells, which are diffuse gastric adenocarcinomas, led to the increased expression of PI3Kp85, pAKT-s473, pGSK3b, and beta-catenin. Conversely, the underexpression of SMARCD3 in SNU601 and SNU668 cells resulted in decreased expression of these signaling molecules.

This study has the following limitations. Although candidates were analyzed in tissues derived from actual patient samples, a detailed survival analysis could not be performed due to the lack of survival data. Therefore, a survival analysis was conducted using the KM plot with a public database. However, the public database did not provide individual patient information, preventing subgroup analysis. Additionally, animal testing was not conducted. However, this is the first study on the role of SMARCD3 in gastric cancer.

In conclusion, the overexpression of SMARCD3 might play a role in promoting EMT in gastric cancer progression, highlighting its involvement in key signaling pathways and their crosstalk, which may offer new avenues for targeted therapeutic interventions. Further research is needed to elucidate the molecular mechanisms underlying the contributions of SMARCD3 to cancer development and progression, with the aim of identifying potential therapeutic targets for precision medicine.

## 5. Conclusions

The overexpression of SMARCD3 might play a role in promoting EMT in gastric cancer progression, highlighting its involvement in key signaling pathways and their crosstalk, which may offer new avenues for targeted therapeutic interventions.

Further research is needed to elucidate the molecular mechanisms underlying the contributions of SMARCD3 to cancer development and progression, with the aim of identifying potential therapeutic targets for precision medicine.

## Data Availability

The data presented in this study are available upon request from the corresponding author.

## Supplementary Materials

The following supporting information can be downloaded at: https://www.mdpi.com/article/10.3390/cancers16122282/s1, Figure S1. Figure 3D. MKN 74 wound healing; Figure S2. Figure 3D. kato3 wound healing; Figure S3. Figure 4A; Figure S4. Figure 4A. s601; Figure S5. Figure 4B. snu 668 MTT; Figure S6. Figure 4B. snu601 MTT; Figure S7. Figure 4D. 668 wound healing; Figure S8. Figure 4D. 601 wound healing; Figure S9. Figure 4E. snu668 invasion; Figure S10. Figure 4E. snu601 invasion; Figure S11. Figure 1A; Figure S12. Figure 1D; Figure S13. Figure 1E; Figure S14. Figure 3A; Figure S15. Figure 3A; Figure S16. Figure 4A; Figure S17. Figure 4A; Figure S18. Figure 6A MKN 74; Figure S19. Figure 6B. KATO III; Figure S20. Figure 6C. SNU601; Figure S21. Figure 6D. SNU668.
