# Supplementary material for: SMARCD3 Overexpression Promotes Epithelial–Mesenchymal Transition in Gastric Cancer
Source: Cancers (Basel). 2024 Jun 20;16(12):2282. doi: 10.3390/cancers16122282 (PMC11201906; doi:10.3390/cancers16122282)
Supplement: Supplementary file 1 [file cancers-16-02282-s001.zip › cancers-3034334-supplementary.pdf]

cancers MDPI

Supplementary Materials: SMARCD3 Overexpression Promotes Epithelial–Mesenchymal Transition in Gastric Cancer

Sun Yi Park, Ji-Ho Park, Jung Wook Yang, Eun-Jung Jung, Young-Tae Ju, Chi-Young Jeong, Ju-Yeon Kim, Taejin Park, Tae-Han Kim, Miyeong Park, Young-Joon Lee and Sang-Ho Jeong

Statistic data of Figure by GraphPad PRISM

Figure S1. Figure 3D. MKN 74 wound healing.

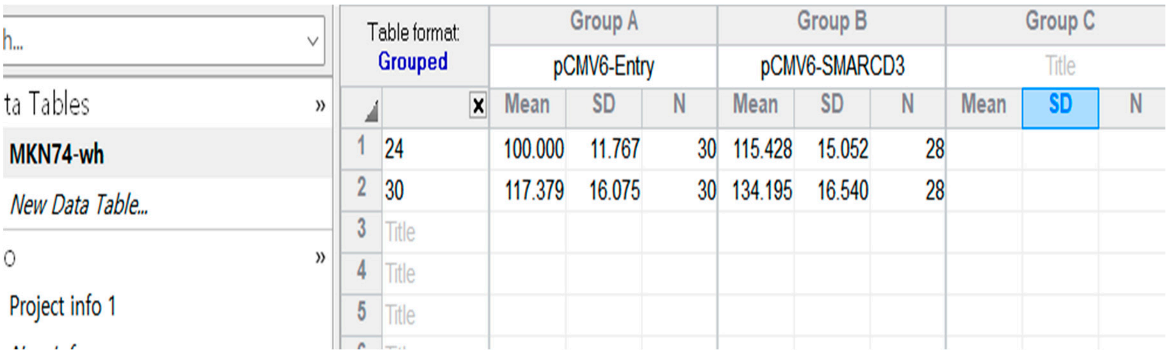

Table format: Grouped	Group A pCMV6-Entry			Group B pCMV6-SMARCD3			Group C Title		
	Mean	SD	N	Mean	SD	N	Mean	SD	N
1 24	100.000	11.767	30	115.428	15.052	28			
2 30	117.379	16.075	30	134.195	16.540	28			
3 Title									
4 Title									
5 Title									

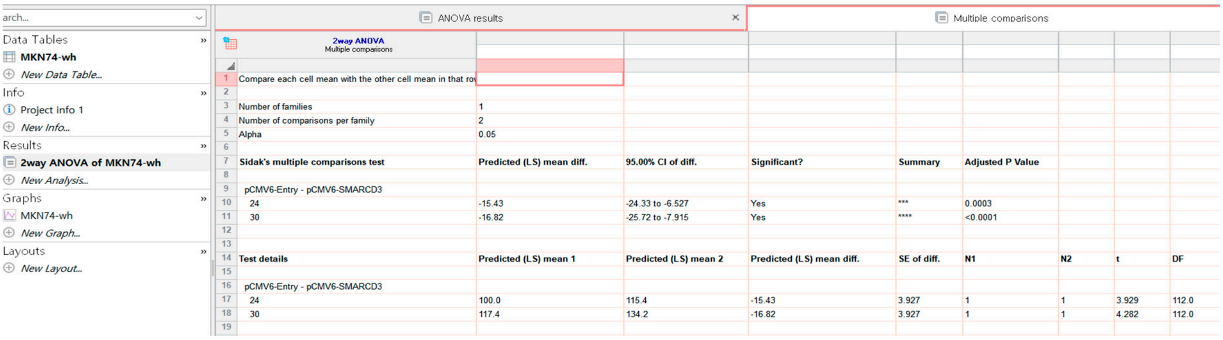

1	Compare each cell mean with the other cell mean in that ro								
2									
3	Number of families	1							
4	Number of comparisons per family	2							
5	Alpha	0.05							
6									
7	**Sidak's multiple comparisons test**	**Predicted (LS) mean diff.**	**95.00% CI of diff.**	**Significant?**	**Summary**	**Adjusted P Value**			
8									
9	pCMV6-Entry - pCMV6-SMARCD3								
10	24	-15.43	-24.33 to -6.527	Yes	***	0.0003			
11	30	-16.82	-25.72 to -7.915	Yes	****	<0.0001			
12									
13									
14	**Test details**	**Predicted (LS) mean 1**	**Predicted (LS) mean 2**	**Predicted (LS) mean diff.**	**SE of diff.**	**N1**	**N2**	**t**	**DF**
15									
16	pCMV6-Entry - pCMV6-SMARCD3								
17	24	100.0	115.4	-15.43	3.927	1	1	3.929	112.0
18	30	117.4	134.2	-16.82	3.927	1	1	4.282	112.0
19									

Figure S2. Figure 3D. kato3 wound healing.

Table format: Grouped	Group A pCMV6-Entry			Group B pCMV6-SMARCD3		
	Mean	SD	N	Mean	SD	N
24	13.50125	24.232	12	11.528	13.536	12
48	18.15205	26.531	12	17.284	14.349	12
96	56.62931	23.694	12	91.386	19.734	12
144	100.00000	38.345	12	149.880	41.617	12

2way ANOVA Multiple comparisons					
Compare each cell mean with the other cell mean in that row					
Number of families	1				
Number of comparisons per family	4				
Alpha	0.05				
Sidak's multiple comparisons test	Mean Diff.	95.00% CI of diff.	Significant?	Summary	Adjusted P Value
pCMV6-Entry - pCMV6-SMARCD3					
24	1.973	-26.06 to 30.01	No	ns	0.9996
48	0.8681	-27.16 to 28.90	No	ns	>0.9999
96	-34.76	-62.79 to -6.724	Yes	**	0.0088
144	-49.88	-77.91 to -21.85	Yes	****	<0.0001

Figure S3. Figure 4A.

Grouped	consiRNA								
	A:1	A:2	A:3	A:4	A:5	A:6	A:7	A:8	A:9
smarcd3	1.00	1.00	1.00	1.00	1.00	1	1	1	1
snail	1.00	1.00	1.00	1.00	1.00				
slug	1.00	1.00	1.00	1.00	1.00				

	Group B smarcd3 siRNA#1								
	B:1	B:2	B:3	B:4	B:5	B:6	B:7	B:8	B:9
smarcd3	0.55	0.46	0.58	0.97	0.55	0.63	0.31	0.35	0.51
snail	0.87	0.59	0.96	0.41	0.66				
slug	0.71	0.61	0.91	1.08	0.49				

	Group C smarcd3 siRNA#2								
	C:1	C:2	C:3	C:4	C:5	C:6	C:7	C:8	C:9
smarcd3	0.33	0.42	0.36	0.64	0.23	0.30	0.49	0.39	0.39
snail	0.78	0.94	0.59	0.76	0.47				
slug	0.59	0.75	0.28	0.62	0.11				

2way ANOVA Multiple comparisons					
Within each row, compare columns (simple effects within rows)					
Number of families	3				
Number of comparisons per family	2				
Alpha	0.05				
Dunnett's multiple comparisons test	Predicted (LS) mean diff.	95.00% CI of diff.	Significant?	Summary	Adjusted P Value
smarcd3					
consiRNA vs. smarcd3 siRNA #1	0.4544	0.2824 to 0.6265	Yes	****	<0.0001
consiRNA vs. smarcd3 siRNA #2	0.6056	0.4335 to 0.7776	Yes	****	<0.0001
snail					
consiRNA vs. smarcd3 siRNA #1	0.3026	0.07178 to 0.5334	Yes	**	0.0085
consiRNA vs. smarcd3 siRNA #2	0.2912	0.06038 to 0.5220	Yes	*	0.0114
slug					
consiRNA vs. smarcd3 siRNA #1	0.2400	0.009178 to 0.4708	Yes	*	0.0405
consiRNA vs. smarcd3 siRNA #2	0.5300	0.2992 to 0.7608	Yes	****	<0.0001

Figure S4. Figure 4A. s601.

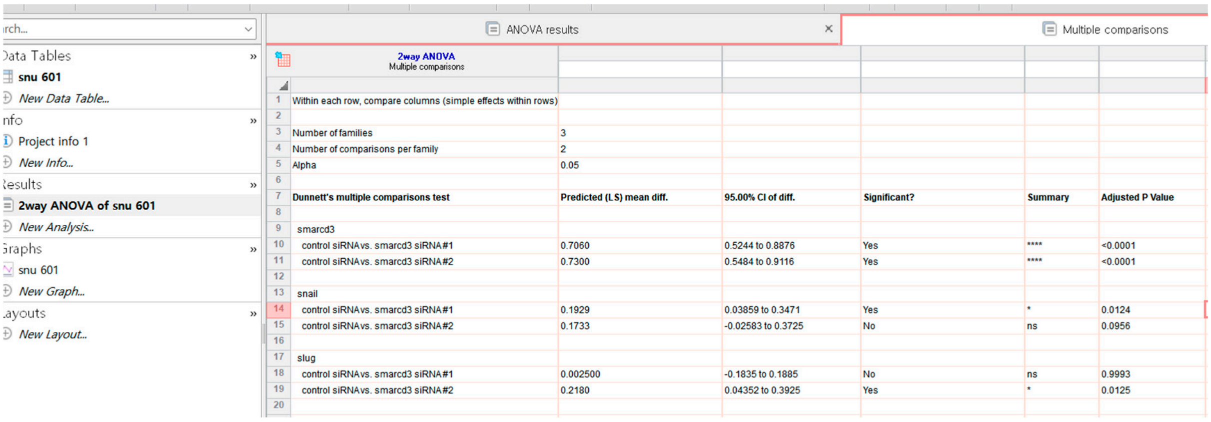

Within each row, compare columns (simple effects within rows)					
Number of families	3				
Number of comparisons per family	2				
Alpha	0.05				
**Dunnett's multiple comparisons test**	**Predicted (LS) mean diff.**	**95.00% CI of diff.**	**Significant?**	**Summary**	**Adjusted P Value**
smarcd3					
control siRNAvs. smarcd3 siRNA#1	0.7060	0.5244 to 0.8876	Yes	****	<0.0001
control siRNAvs. smarcd3 siRNA#2	0.7300	0.5484 to 0.9116	Yes	****	<0.0001
snail					
control siRNAvs. smarcd3 siRNA#1	0.1929	0.03859 to 0.3471	Yes	*	0.0124
control siRNAvs. smarcd3 siRNA#2	0.1733	-0.02583 to 0.3725	No	ns	0.0956
slug					
control siRNAvs. smarcd3 siRNA#1	0.002500	-0.1835 to 0.1885	No	ns	0.9993
control siRNAvs. smarcd3 siRNA#2	0.2180	0.04352 to 0.3925	Yes	*	0.0125

Figure S5. Figure 4B. snu 668 MTT.

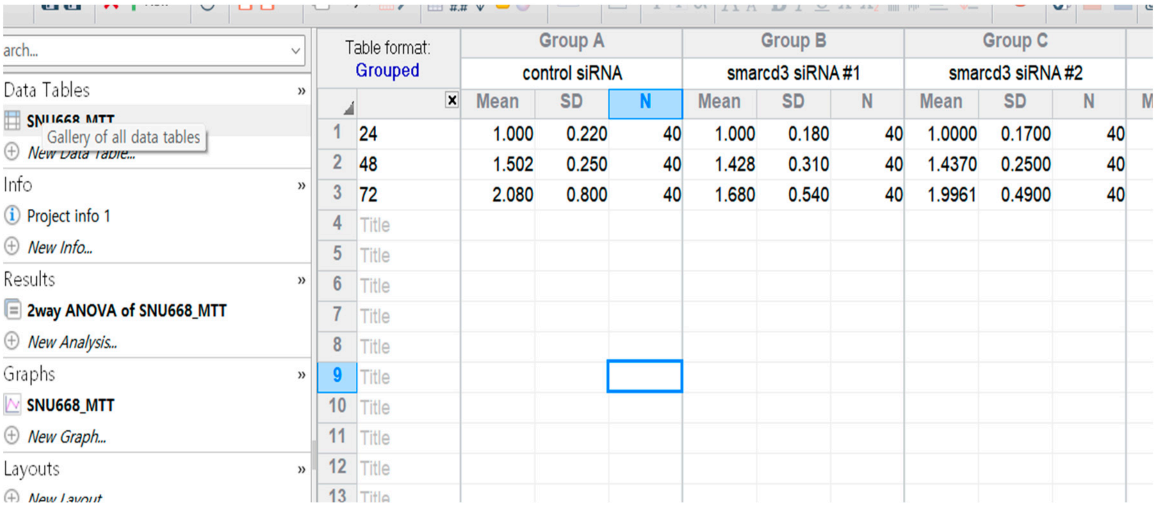

	Group A			Group B			Group C		
	control siRNA			smarcd3 siRNA #1			smarcd3 siRNA #2		
	Mean	SD	N	Mean	SD	N	Mean	SD	N
24	1.000	0.220	40	1.000	0.180	40	1.0000	0.1700	40
48	1.502	0.250	40	1.428	0.310	40	1.4370	0.2500	40
72	2.080	0.800	40	1.680	0.540	40	1.9961	0.4900	40

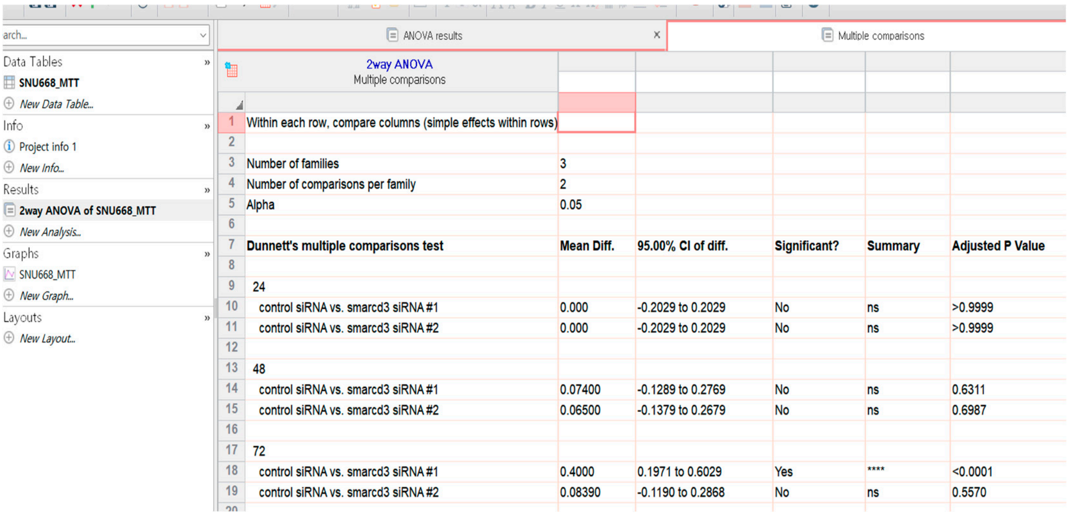

Within each row, compare columns (simple effects within rows)					
Number of families	3				
Number of comparisons per family	2				
Alpha	0.05				
**Dunnett's multiple comparisons test**	**Mean Diff.**	**95.00% CI of diff.**	**Significant?**	**Summary**	**Adjusted P Value**
24					
control siRNA vs. smarcd3 siRNA #1	0.000	-0.2029 to 0.2029	No	ns	>0.9999
control siRNA vs. smarcd3 siRNA #2	0.000	-0.2029 to 0.2029	No	ns	>0.9999
48					
control siRNA vs. smarcd3 siRNA #1	0.07400	-0.1289 to 0.2769	No	ns	0.6311
control siRNA vs. smarcd3 siRNA #2	0.06500	-0.1379 to 0.2679	No	ns	0.6987
72					
control siRNA vs. smarcd3 siRNA #1	0.4000	0.1971 to 0.6029	Yes	****	<0.0001
control siRNA vs. smarcd3 siRNA #2	0.08390	-0.1190 to 0.2868	No	ns	0.5570

Figure S6. Figure 4B. snu601 MTT.

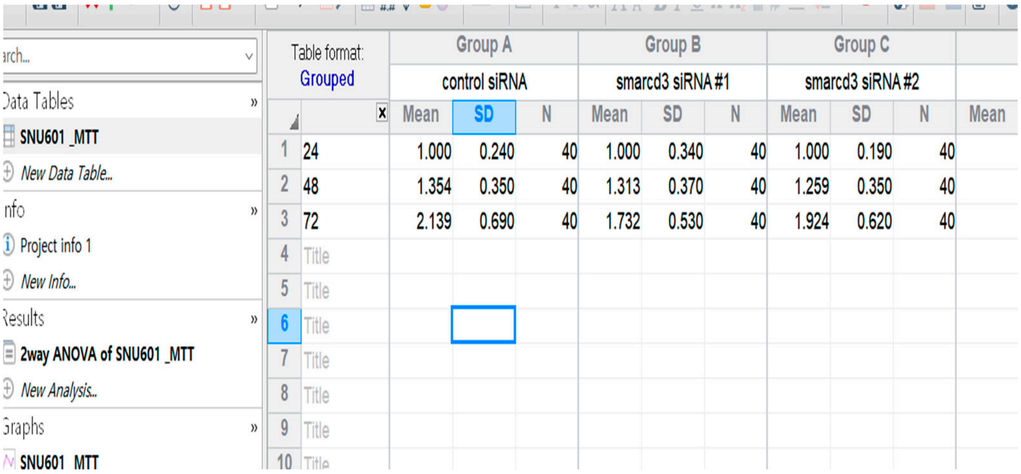

Table format: Grouped	Group A: control siRNA			Group B: smarcd3 siRNA #1			Group C: smarcd3 siRNA #2		
	Mean	SD	N	Mean	SD	N	Mean	SD	N
24	1.000	0.240	40	1.000	0.340	40	1.000	0.190	40
48	1.354	0.350	40	1.313	0.370	40	1.259	0.350	40
72	2.139	0.690	40	1.732	0.530	40	1.924	0.620	40

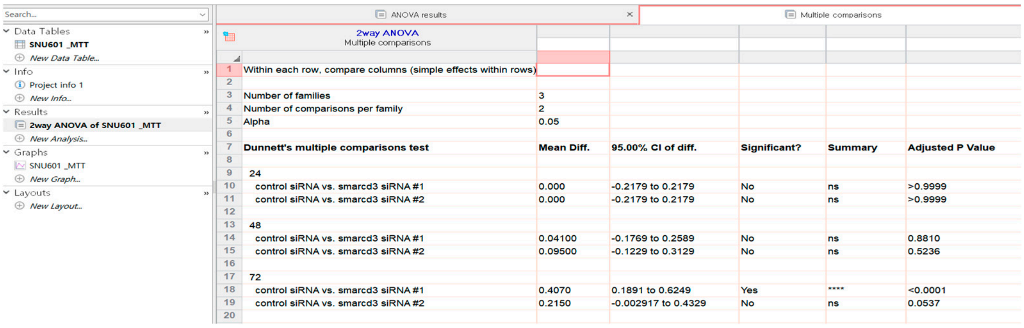

2way ANOVA
Multiple comparisons

Within each row, compare columns (simple effects within rows)					
Number of families	3				
Number of comparisons per family	2				
Alpha	0.05				
**Dunnett's multiple comparisons test**	**Mean Diff.**	**95.00% CI of diff.**	**Significant?**	**Summary**	**Adjusted P Value**
24					
control siRNA vs. smarcd3 siRNA #1	0.000	-0.2179 to 0.2179	No	ns	>0.9999
control siRNA vs. smarcd3 siRNA #2	0.000	-0.2179 to 0.2179	No	ns	>0.9999
48					
control siRNA vs. smarcd3 siRNA #1	0.04100	-0.1769 to 0.2589	No	ns	0.8810
control siRNA vs. smarcd3 siRNA #2	0.09500	-0.1229 to 0.3129	No	ns	0.5236
72					
control siRNA vs. smarcd3 siRNA #1	0.4070	0.1891 to 0.6249	Yes	****	<0.0001
control siRNA vs. smarcd3 siRNA #2	0.2150	-0.002917 to 0.4329	No	ns	0.0537

Figure S7. Figure 4D. 668 wound healing.

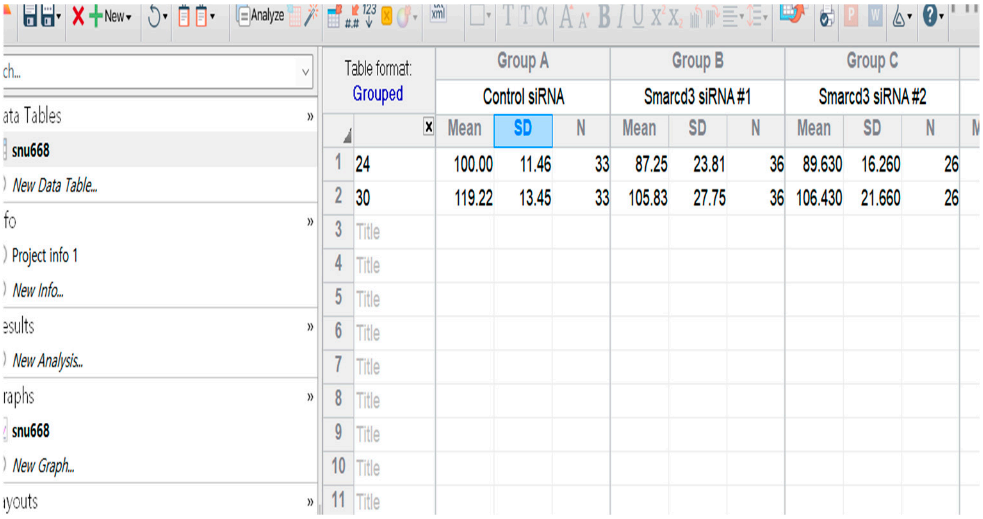

	Table format: Grouped	Group A Control siRNA			Group B Smarcd3 siRNA #1			Group C Smarcd3 siRNA #2		
		Mean	SD	N	Mean	SD	N	Mean	SD	N
1	24	100.00	11.46	33	87.25	23.81	36	89.630	16.260	26
2	30	119.22	13.45	33	105.83	27.75	36	106.430	21.660	26
3	Title									
4	Title									
5	Title									
6	Title									
7	Title									
8	Title									
9	Title									
10	Title									
11	Title									

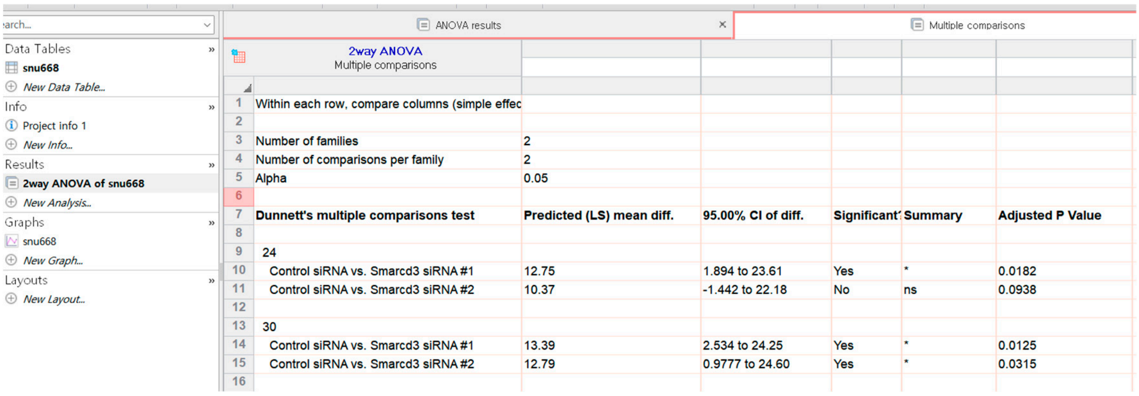

	2way ANOVA Multiple comparisons					
1	Within each row, compare columns (simple effec					
2						
3	Number of families	2				
4	Number of comparisons per family	2				
5	Alpha	0.05				
6						
7	Dunnett's multiple comparisons test	Predicted (LS) mean diff.	95.00% CI of diff.	Significant?	Summary	Adjusted P Value
8						
9	24					
10	Control siRNA vs. Smarcd3 siRNA #1	12.75	1.894 to 23.61	Yes	*	0.0182
11	Control siRNA vs. Smarcd3 siRNA #2	10.37	-1.442 to 22.18	No	ns	0.0938
12						
13	30					
14	Control siRNA vs. Smarcd3 siRNA #1	13.39	2.534 to 24.25	Yes	*	0.0125
15	Control siRNA vs. Smarcd3 siRNA #2	12.79	0.9777 to 24.60	Yes	*	0.0315
16						

Figure S8. Figure 4D. 601 wound healing.

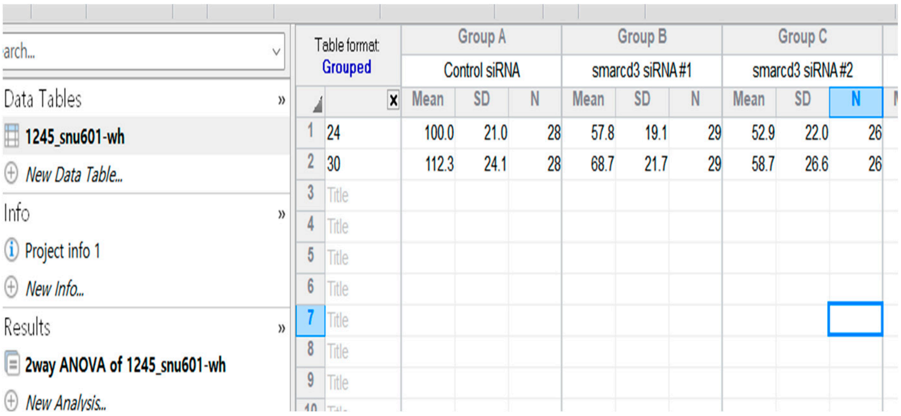

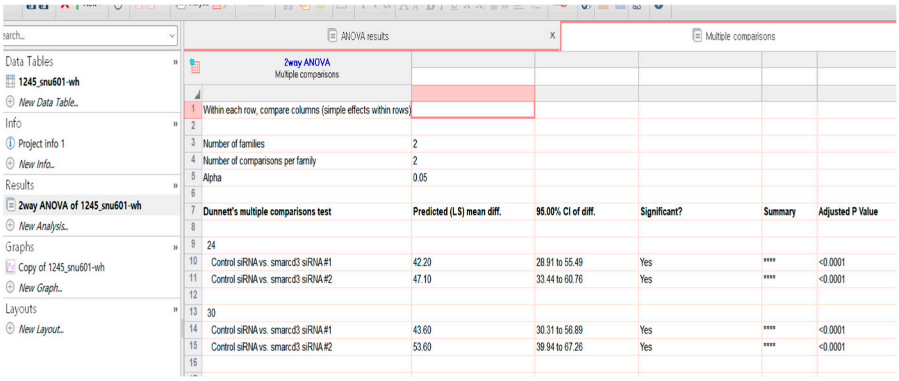

Figure S9. Figure 4E. snu668 invasion.

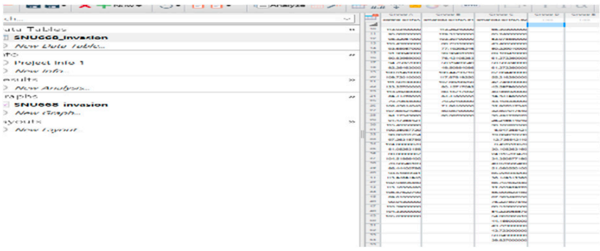

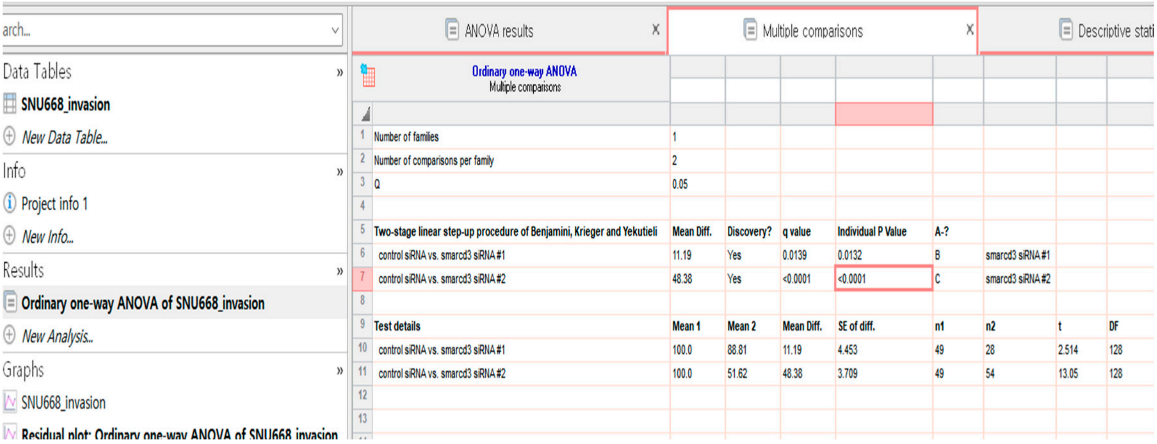

Figure S10. Figure 4E. snu601 invasion.

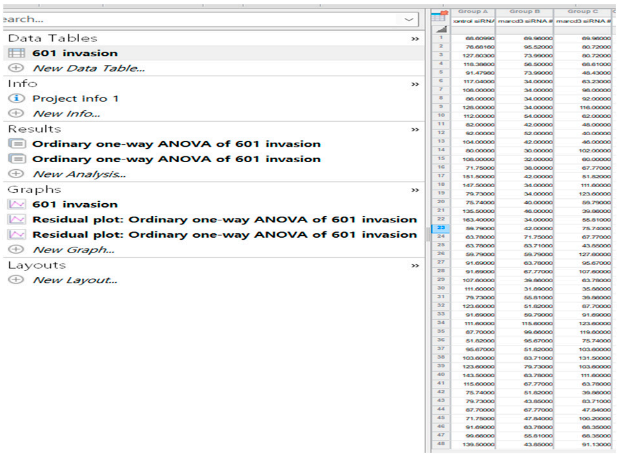

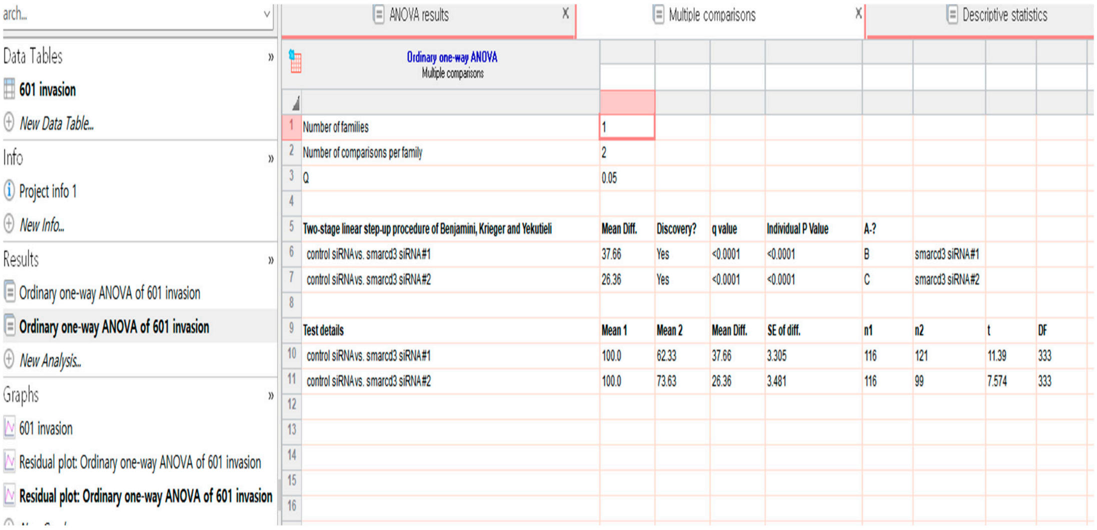

	Ordinary one-way ANOVA Multiple comparisons								
1	Number of families	1							
2	Number of comparisons per family	2							
3	Q	0.05							
4									
5	Two-stage linear step-up procedure of Benjamini, Krieger and Yekutieli	Mean Diff.	Discovery?	q value	Individual P Value	A-?			
6	control siRNA vs. smarcd3 siRNA#1	37.66	Yes	<0.0001	<0.0001	B	smarcd3 siRNA#1		
7	control siRNA vs. smarcd3 siRNA#2	26.36	Yes	<0.0001	<0.0001	C	smarcd3 siRNA#2		
8									
9	Test details	Mean 1	Mean 2	Mean Diff.	SE of diff.	n1	n2	t	DF
10	control siRNA vs. smarcd3 siRNA#1	100.0	62.33	37.66	3.305	116	121	11.39	333
11	control siRNA vs. smarcd3 siRNA#2	100.0	73.63	26.36	3.481	116	99	7.574	333
12									
13									
14									
15									
16									

Western Blot Original Figures

We used protein size marker Thermo(#26619) and GenDEPOT(P8502)

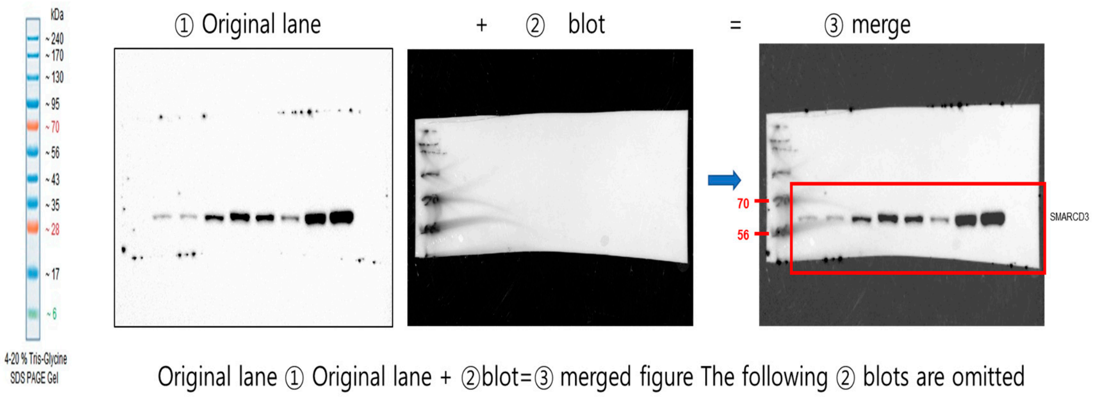

Figure S11. Fig 1. A

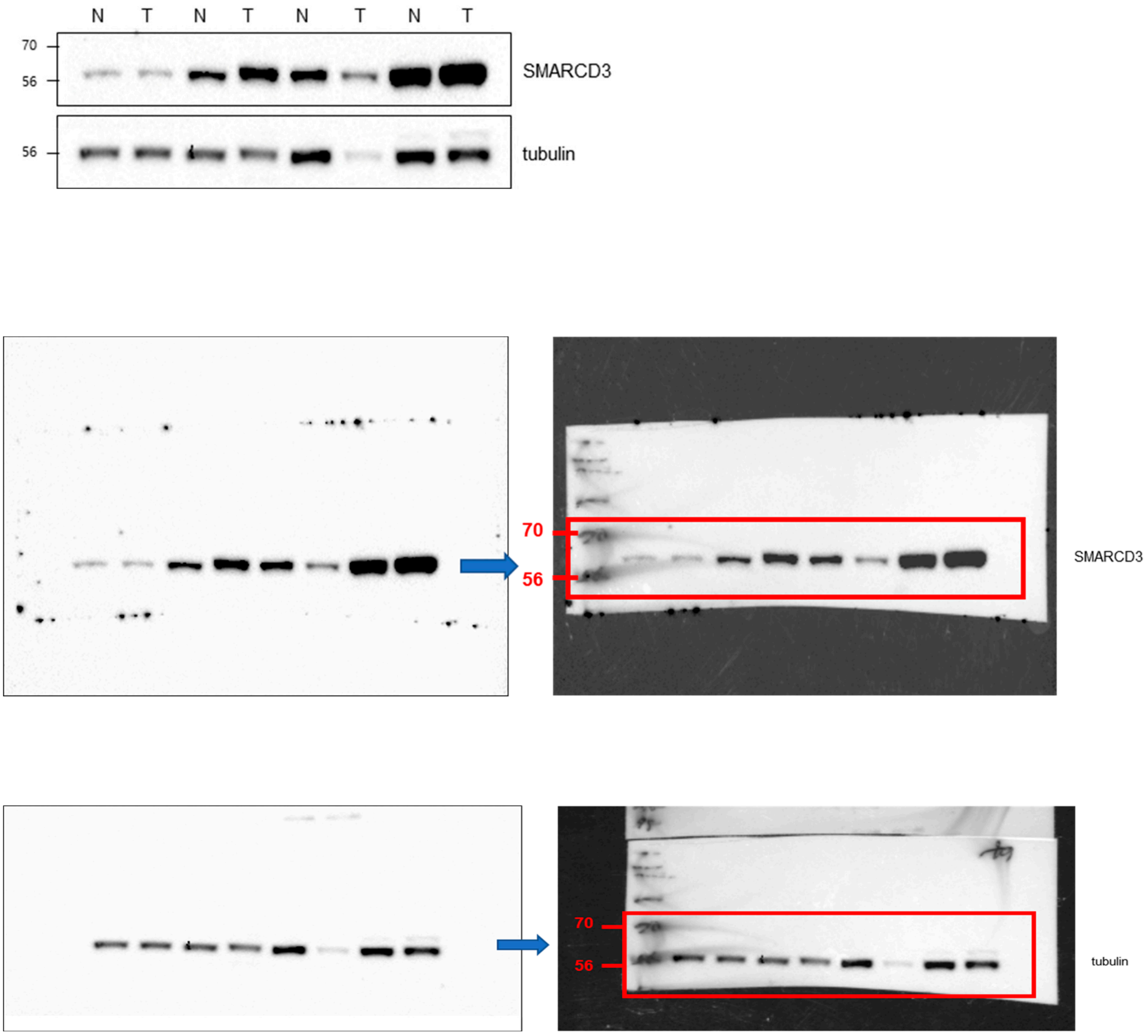

Figure S12. Fig 1. D

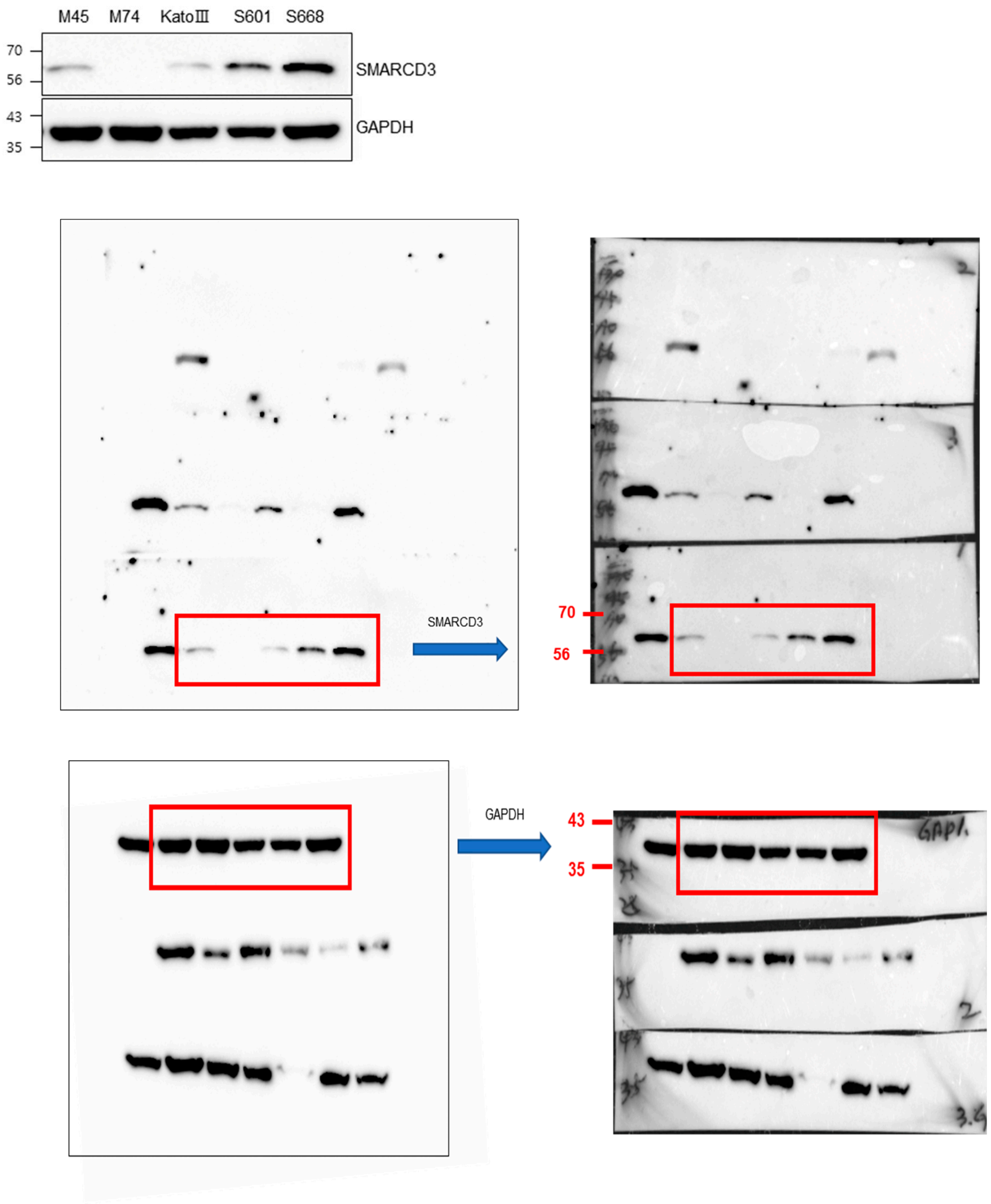

Figure S13. Fig 1. E

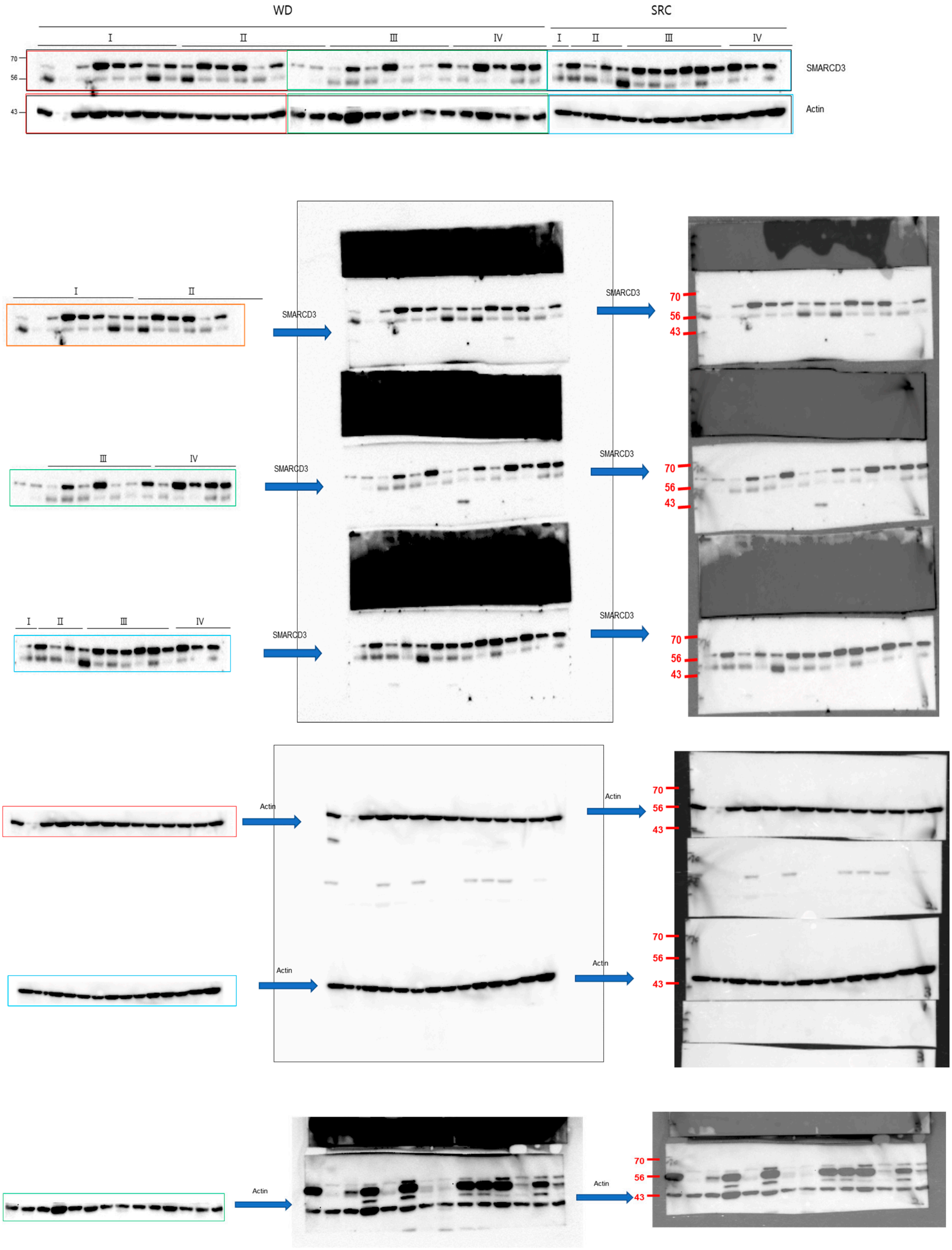

Figure S14. Fig 3. A

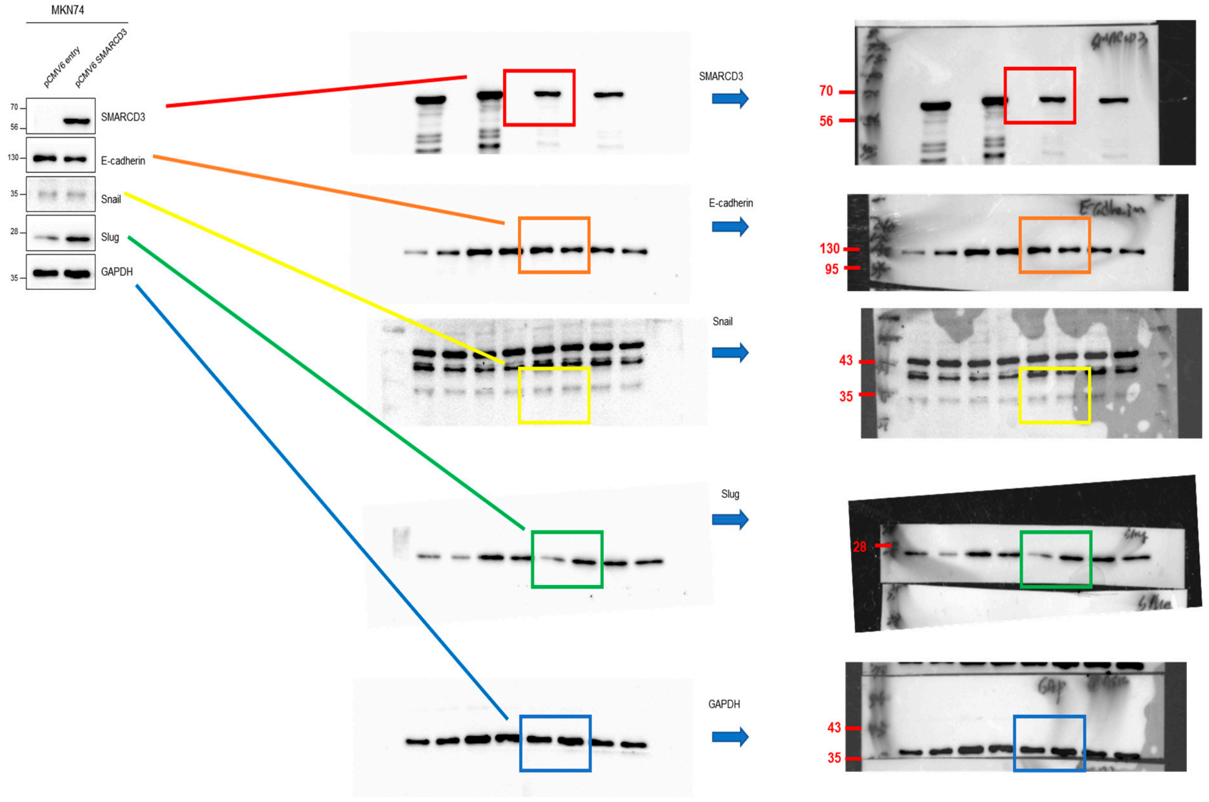

Figure S15. Fig 3. A

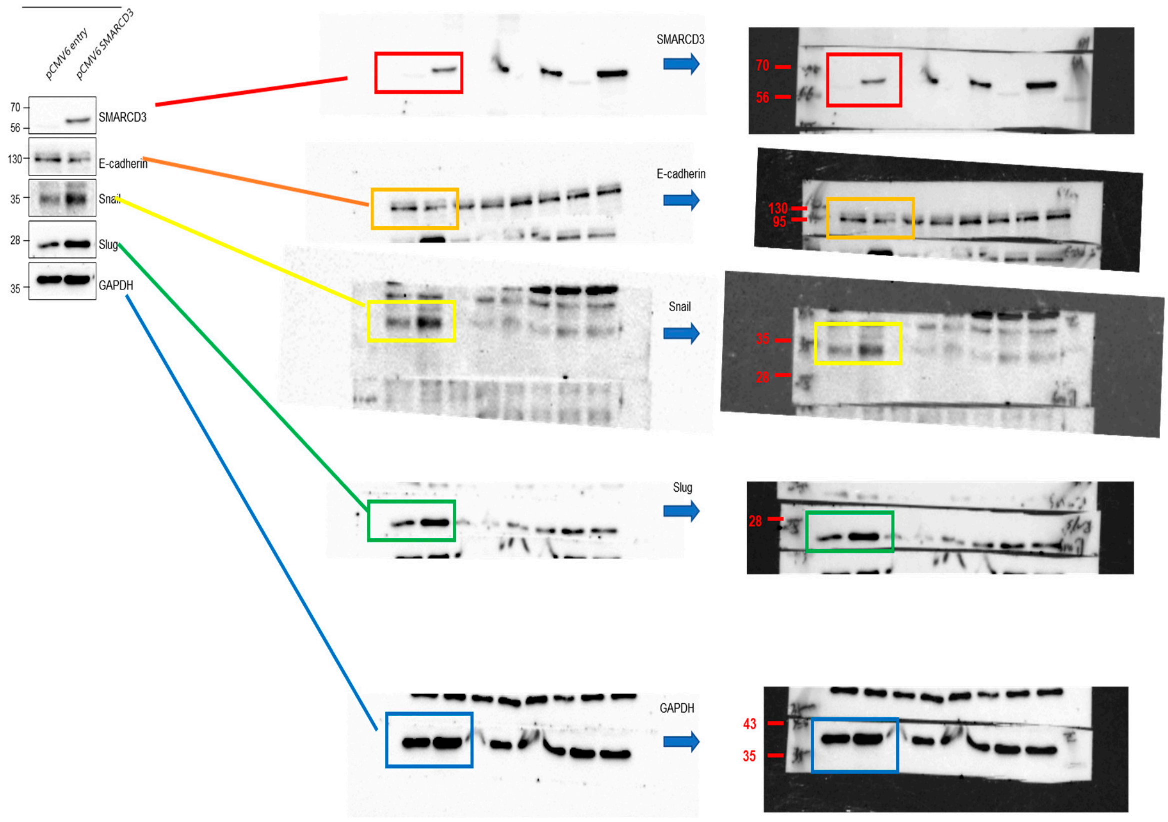

Figure S16. Fig 4. A

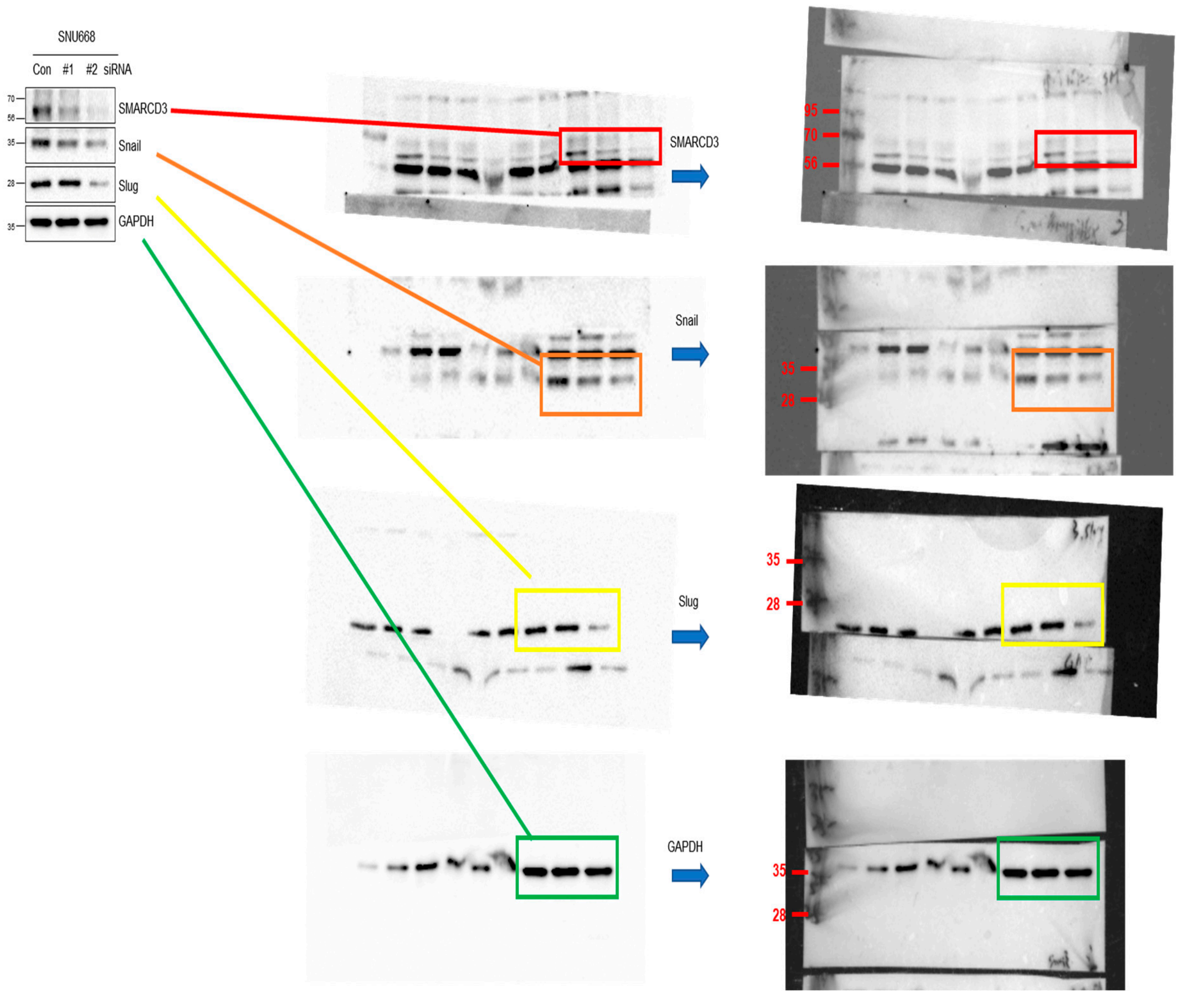

Figure S17. Fig 4. A

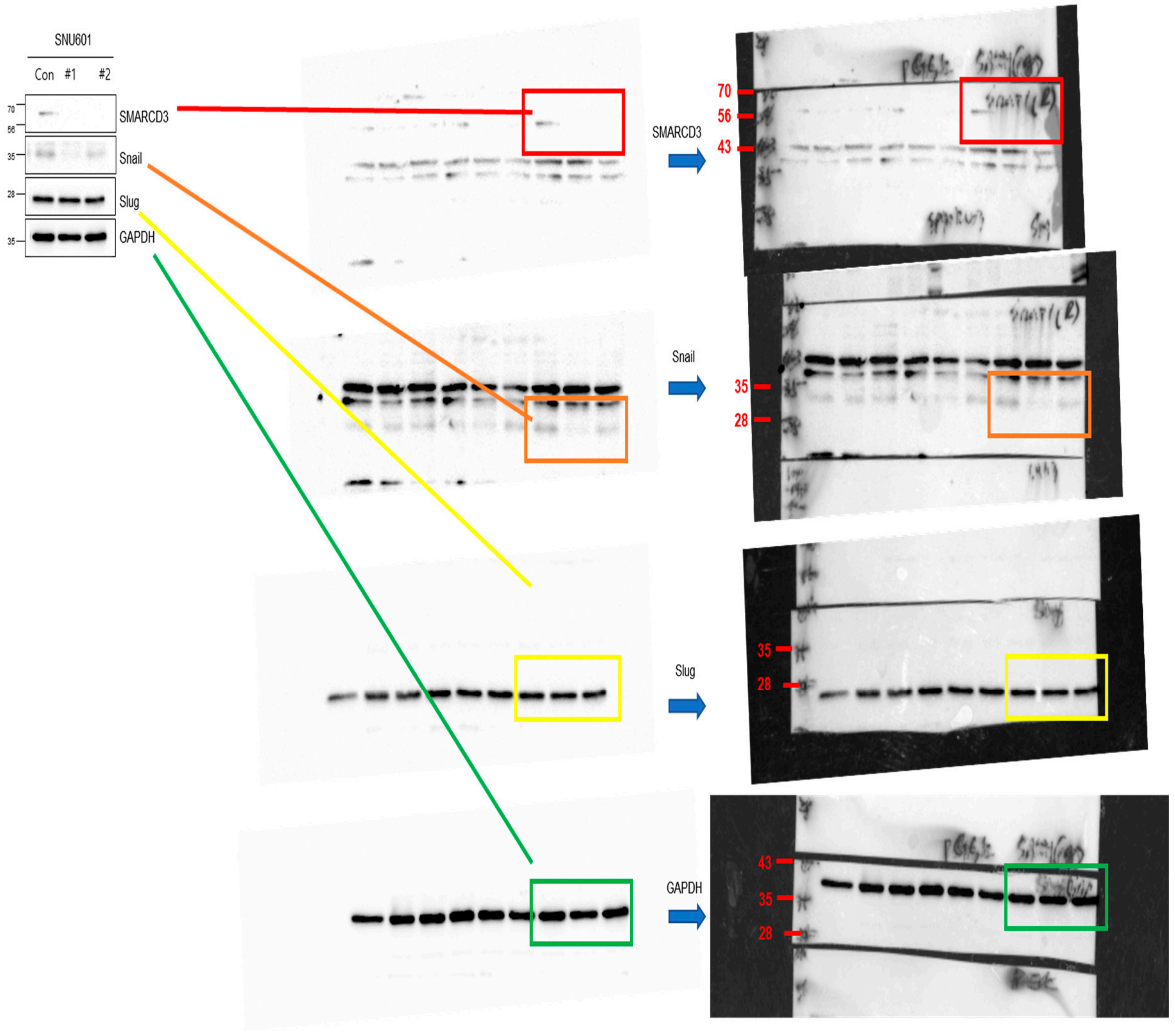

Figure S18. Fig 6. A MKN 74

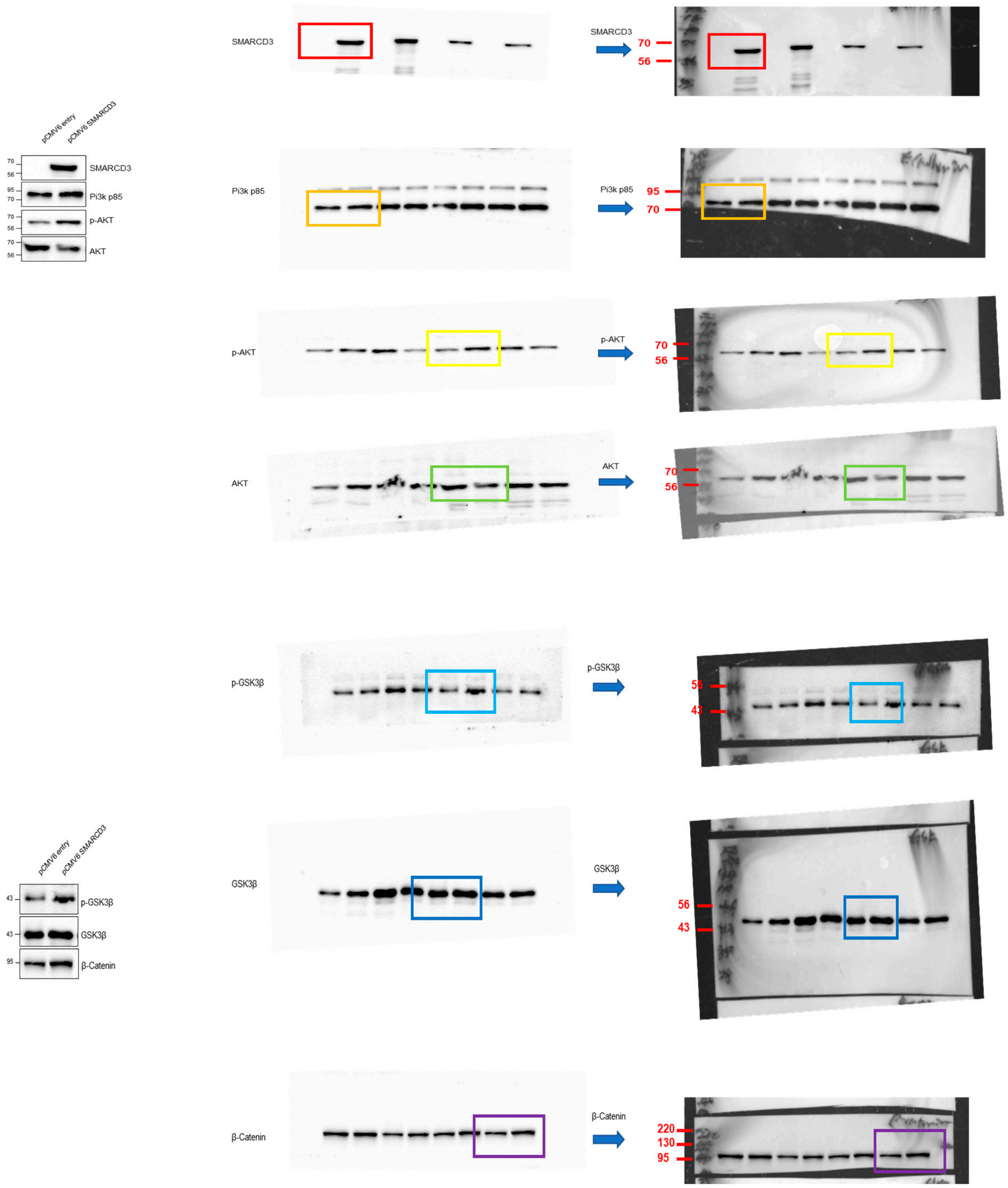

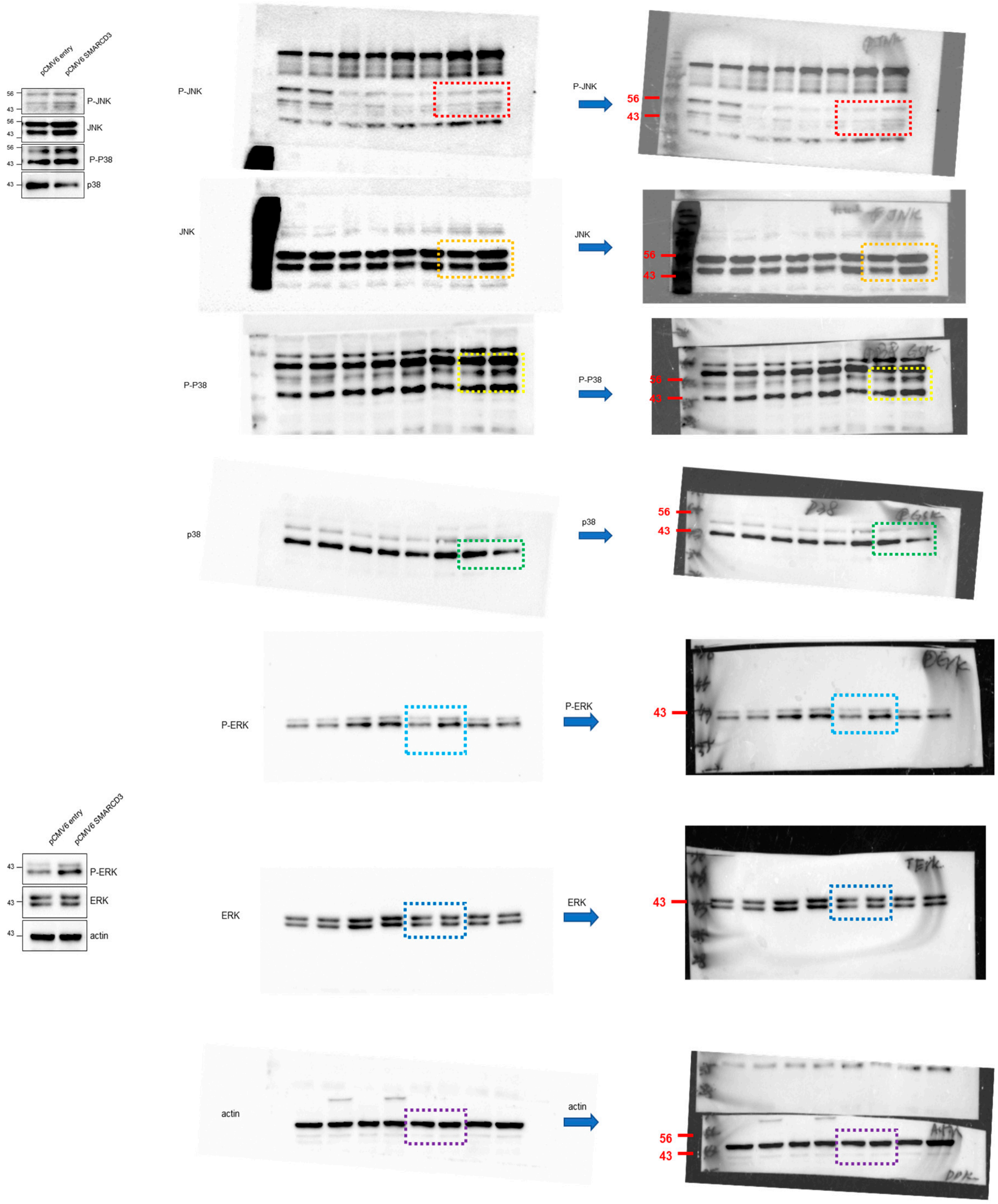
pCMV6 entry
pCMV6 SMARCD3
P-JNK
JNK
P-P38
p38
P-ERK
ERK
actin
56
43

Figure S19. Fig 6. B. KATOIII

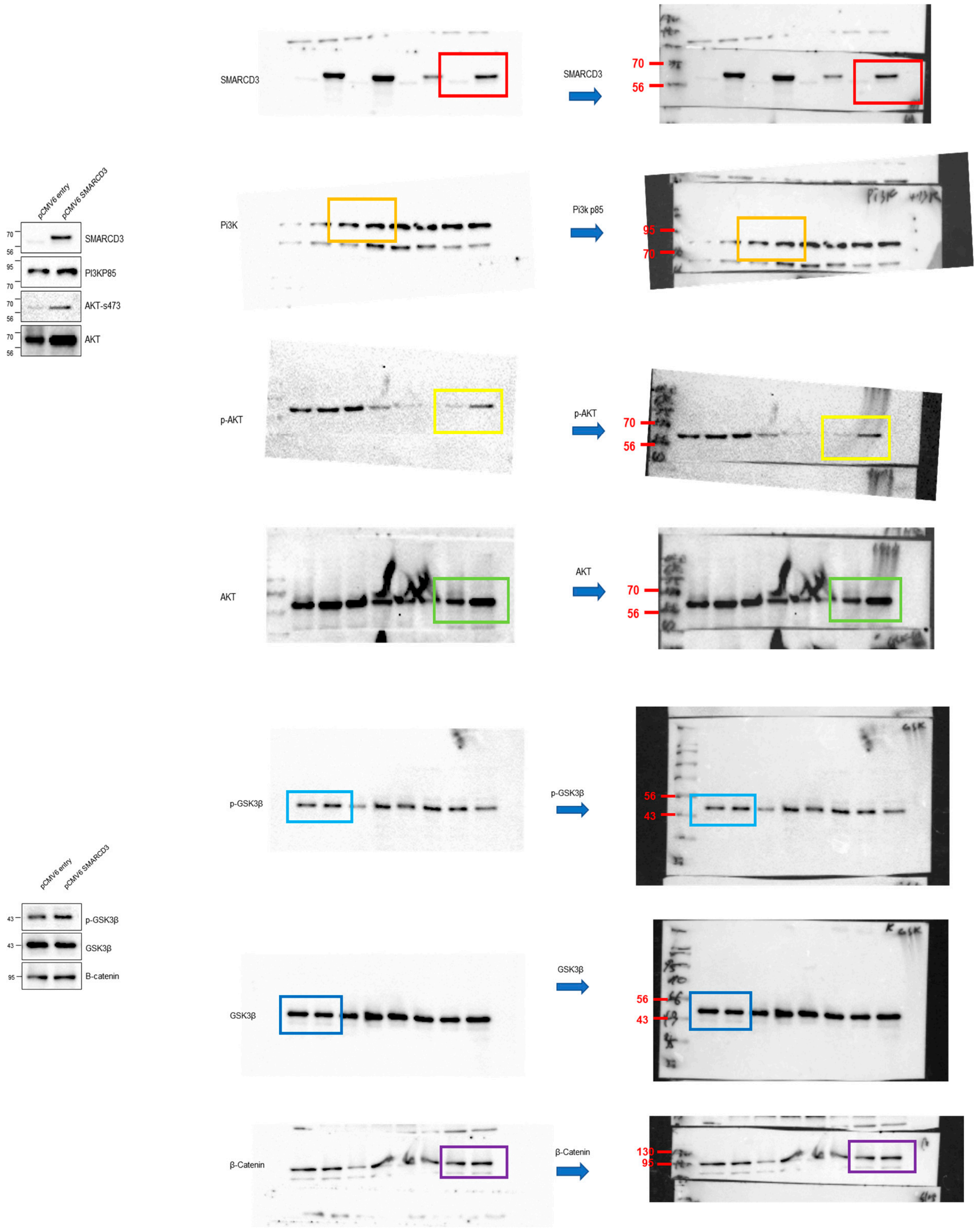

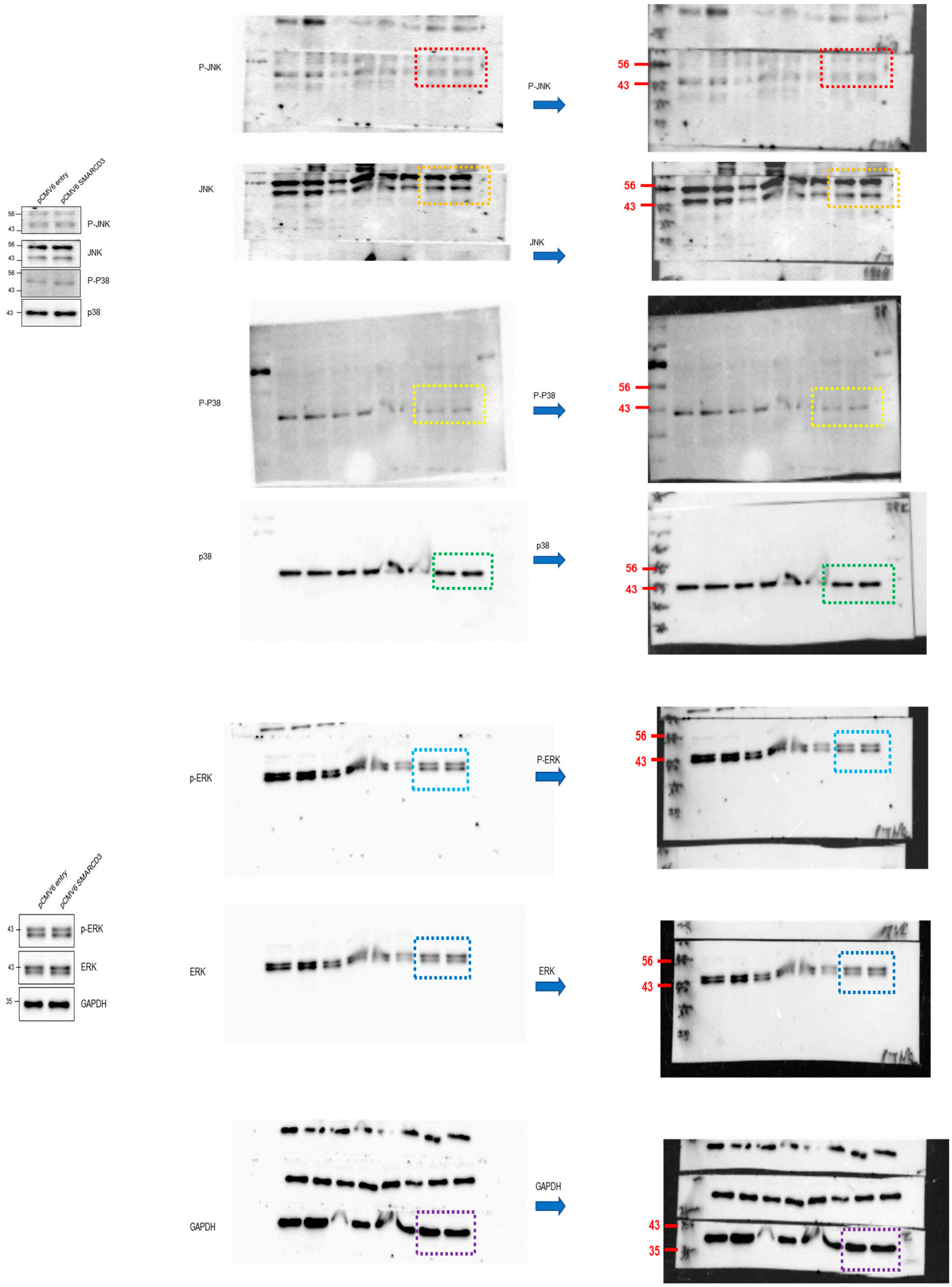
pCMV6 entry
pCMV6 SMARCD3
P-JNK
JNK
P-P38
p38
p-ERK
ERK
GAPDH
56
43
35

Figure S20. Fig 6. C. SNU601

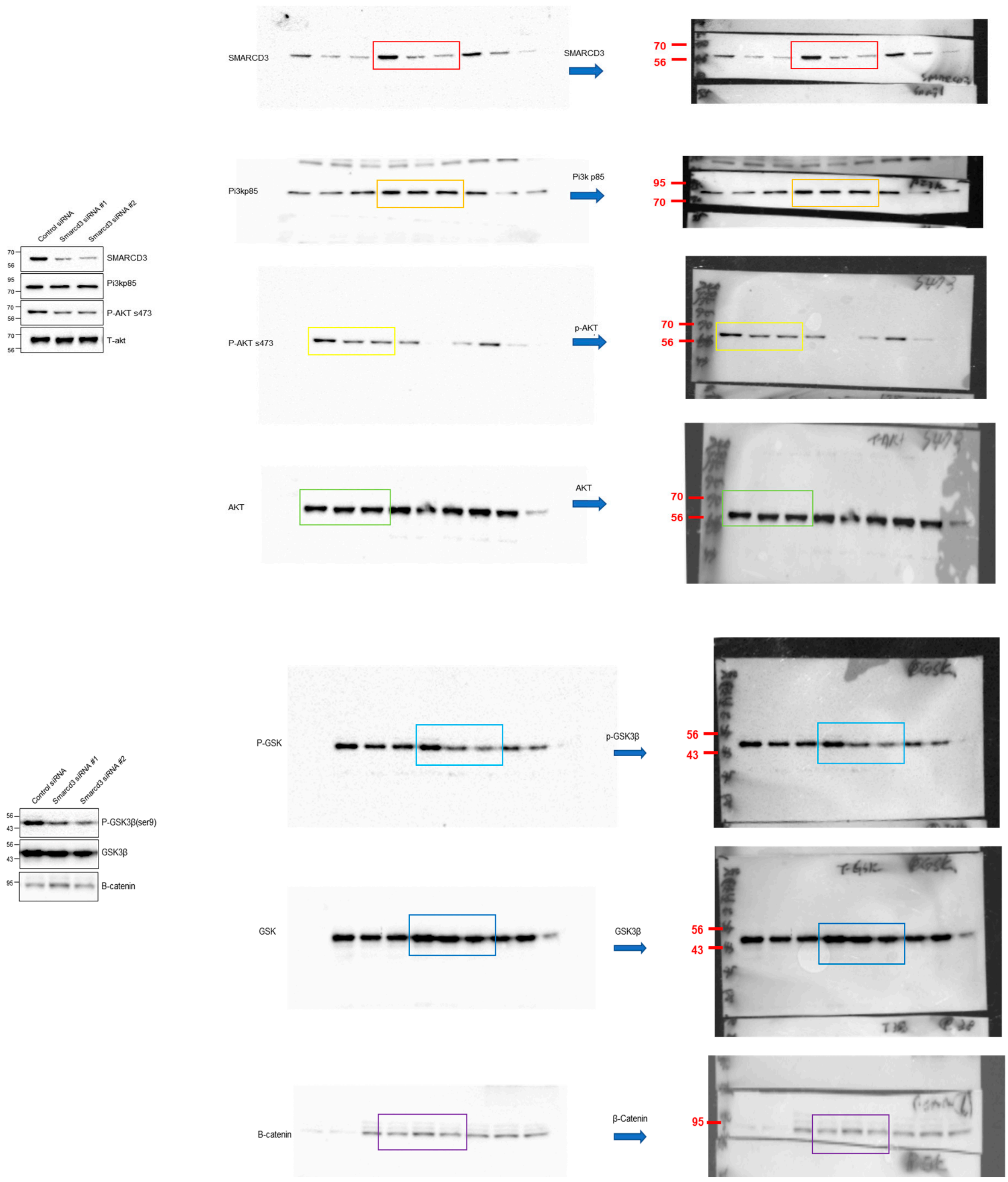

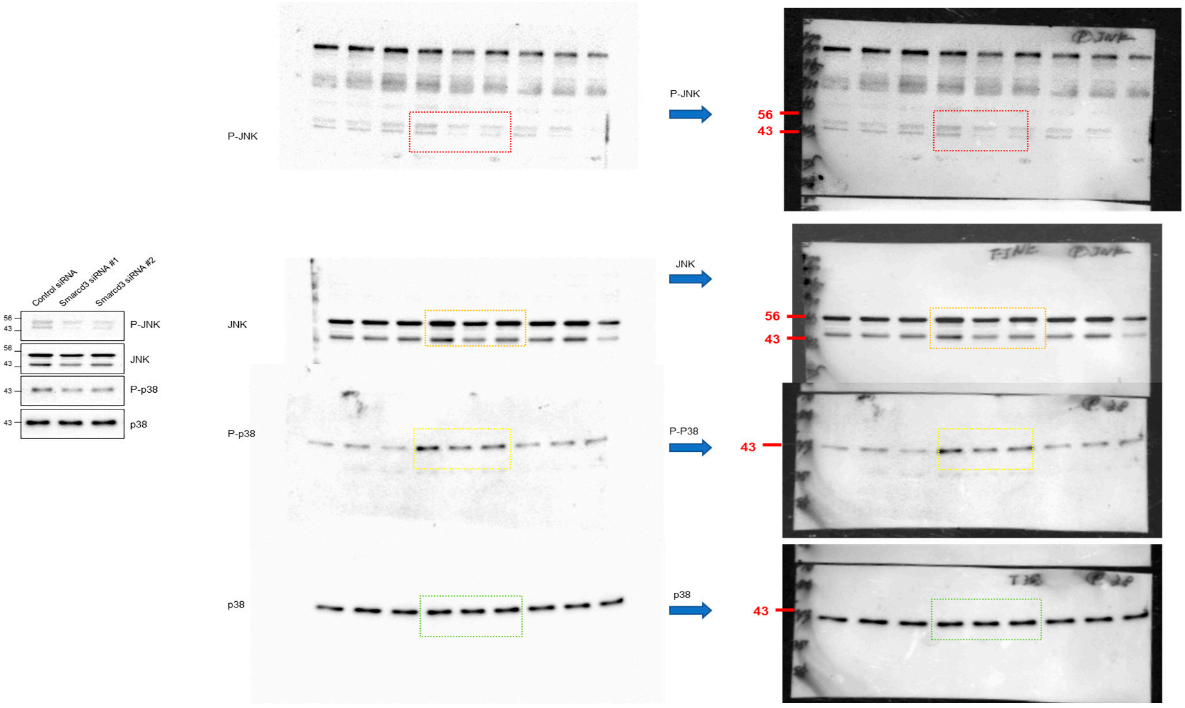
Control siRNA
Smarcd3 siRNA #1
Smarcd3 siRNA #2
P-JNK
JNK
P-p38
p38
56
43
P-P38

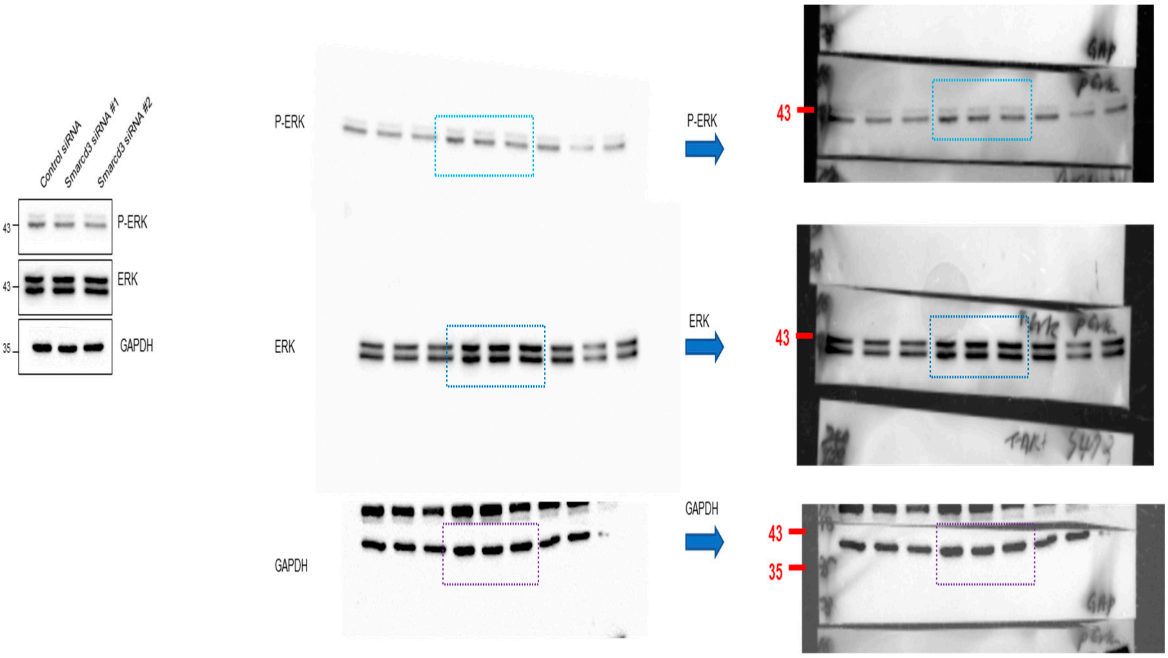
Control siRNA
Smarcd3 siRNA #1
Smarcd3 siRNA #2
P-ERK
ERK
GAPDH
43
35

Figure S21. Fig 6. D. SNU668

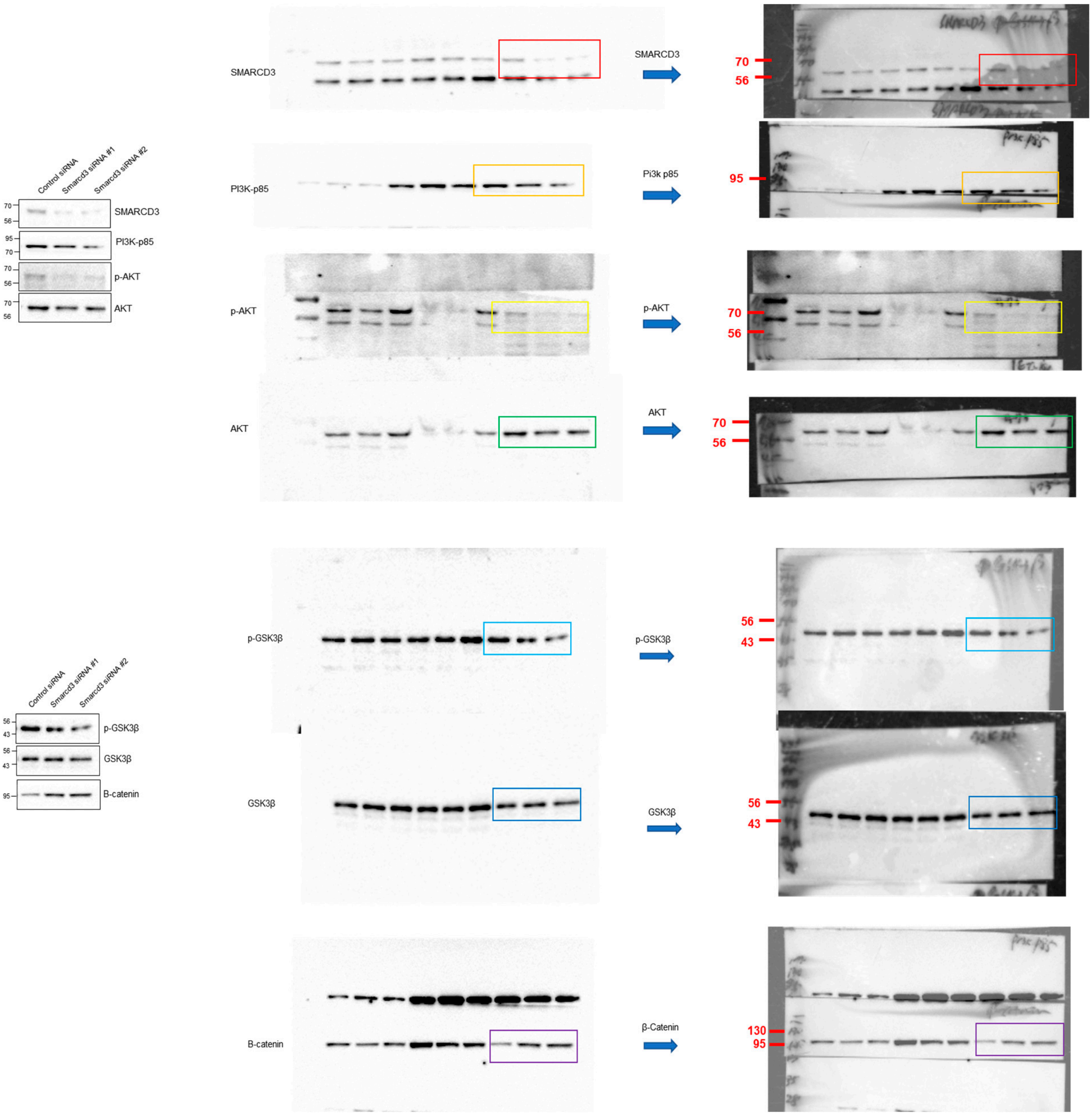

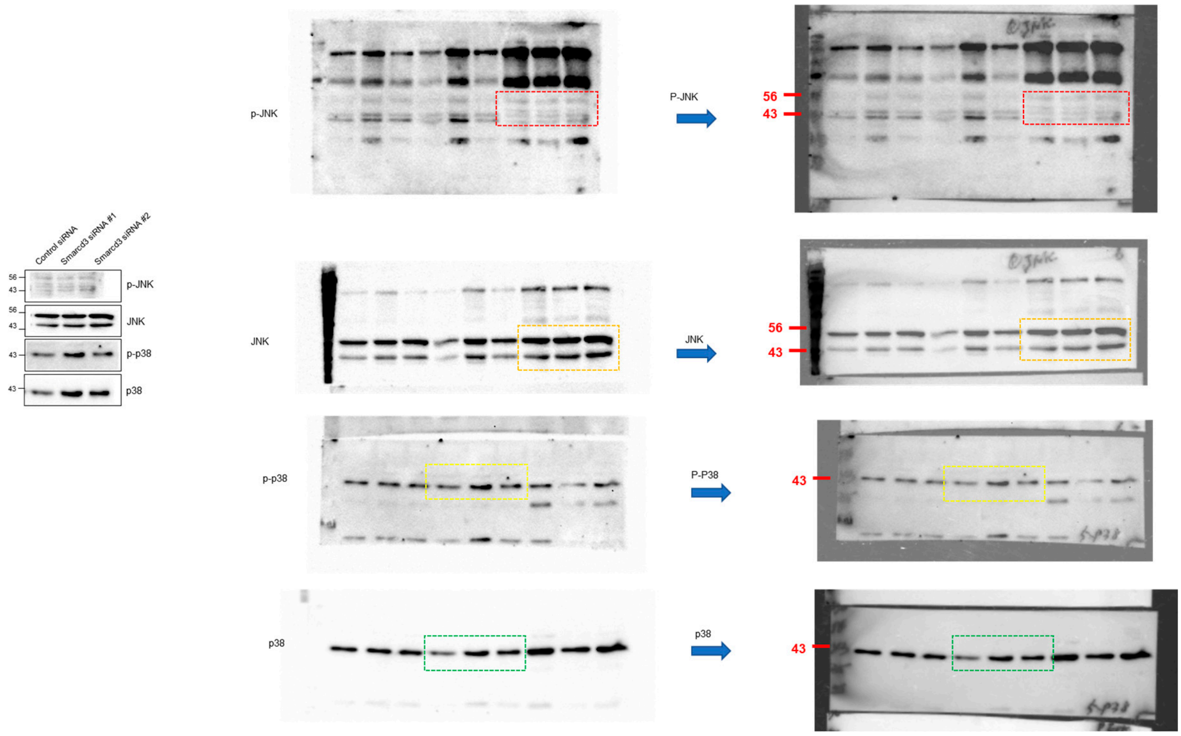
Control siRNA
Smarcd3 siRNA #1
Smarcd3 siRNA #2
56
43
p-JNK
56
43
JNK
43
p-p38
43
p38
p-JNK
P-JNK
56
43
JNK
JNK
56
43
p-p38
P-P38
43
p38
p38
43

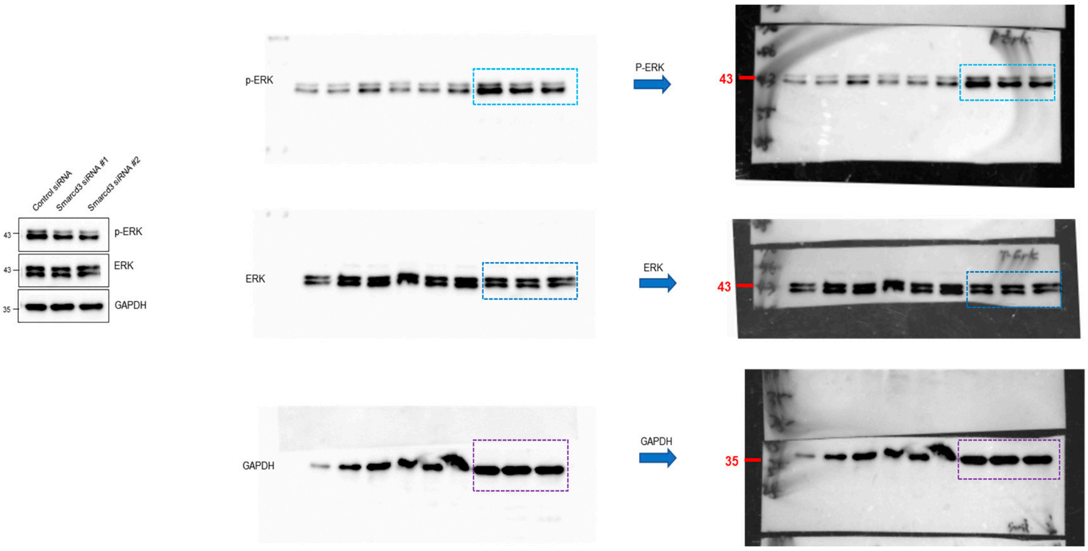
Control siRNA
Smarcd3 siRNA #1
Smarcd3 siRNA #2
43
p-ERK
43
ERK
35
GAPDH
p-ERK
P-ERK
43
ERK
ERK
43
GAPDH
GAPDH
35
